# Molecular Network Approach to Anisotropic Ising Lattices: Parsing Magnetization Dynamics in Er^3+^ Systems with 0–3-Dimensional Spin Interactivity

**DOI:** 10.1021/jacs.3c08946

**Published:** 2023-09-29

**Authors:** Angelica P. Orlova, Maxwell S. Varley, Maximilian G. Bernbeck, Kyle M. Kirkpatrick, Philip C. Bunting, Milan Gembicky, Jeffrey D. Rinehart

**Affiliations:** Department of Chemistry and Biochemistry, University of California, San Diego, La Jolla, California 92093, United States

## Abstract

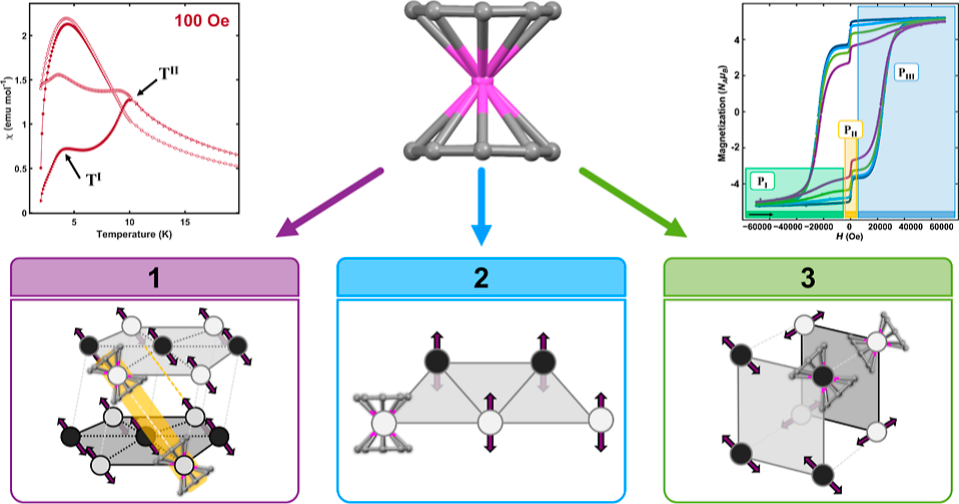

We present a wide-ranging interrogation of the border between single-molecule and solid-state magnetism through a study of erbium-based Ising-type magnetic compounds with a fixed magnetic unit, using three different charge-balancing cations as the means to modulate the crystal packing environment. Properties rooted in the isolated spin Hamiltonian remain fixed, yet careful observation of the dynamics reveals the breakdown of this approximation in a number of interesting ways. First, differences in crystal packing lead to a striking 3 orders of magnitude suppression in magnetic relaxation rates, indicating a rich interplay between intermolecular interactions governed by the anisotropic Ising lattice stabilization and localized slow magnetic relaxation driven by the spin-forbidden nature of quantum tunneling of the f-electron-based magnetization. By means of diverse and rigorous physical methods, including temperature-dependent X-ray crystallography, field, temperature, and time-dependent magnetometry, and the application of a new magnetization fitting technique to quantify the magnetic susceptibility peakshape, we are able to construct a more nuanced view of the role nonzero-dimensional interactions can play in what are predominantly considered zero-dimensional magnetic materials. Specifically, we use low field susceptibility and virgin-curve analysis to isolate metamagnetic spin-flip transitions in each system with a field strength corresponding to the expected strength of the internal dipole–dipole lattice. This behavior is vital to a complete interpretation of the dynamics and is likely common for systems with such high anisotropy. This collective interactivity opens a new realm of possibility for molecular magnetic materials, where their unprecedented localized anisotropy is the determining factor in building higher dimensionality.

## Introduction

The design of functional magnetic materials has been a long-standing endeavor in the realm of materials science. While magnetic behavior is often associated with the ordering behavior of bulk materials, current technological trends in miniaturization, spin-based electronics, and the harnessing of quantum information have expanded the scope of the possibility of what can constitute a functional material to a far broader range of time, size, and interaction strength. The field of single-molecule magnetism has revealed the inherent molecular-level magnetism possible through careful synthetic design of molecules.^1−5^ Over the last 40 years, the effects of quantization, exchange coupling, and spatial anisotropy on the time, temperature, and field dependence of spin polarization under both equilibrium and nonequilibrium conditions have all been targeted for study. Single-molecule magnets (SMMs)^6−10^ possess remarkable properties such as a time-dependent spin memory effect similar to a superparamagnet, except acting via quantized states. This ability to retain and modulate spin at the molecular level with chemically tailored property control has generated substantial interest of a fundamental as well as applied nature in alternative information storage mechanisms, quantum computing, and molecular spintronics, among other promising applications.^11−16^ One of the largest challenges to SMM implementation is the poor translation from the behavior of SMMs in isolation to SMMs interacting with each other or other external degrees of freedom. In actuality, all SMMs are intrinsically dependent on their environment, and any application must recognize, and ideally leverage, this crucial point.

To continue to advance SMMs within a functional materials context, it becomes imperative to explore the profoundly complex connection between single-molecule properties and their surrounding environment. This includes the connection between the spin system and vibronic degrees of freedom,^17−21^ coupling to external electromagnetic radiation,^22−26^ as well as coupling to the internal magnetic fields generated by the arrangement of SMM magnetic moments. The potential for local magnetic moments to influence global magnetic dynamics has not gone unnoticed,^27−29^ largely in the solid-state literature of metallic, ionic, and covalent solids, where discourse on correlated interactions is imperative due to their strength. In molecular solids, correlated interactions are far less discussed, yet on the energy scale of magnetization dynamics, they can play an important role, especially in samples with small crystallites and under nonequilibrium conditions. SMMs containing a single lanthanide ion designed to induce maximum axial anisotropy (often a Dy^3+^/Er^3+^ ion with ground state |*J* = 15/2, *m*_*J*_ = ± 15/2⟩) possess some of the largest and most anisotropic angular momenta possible on a per-ion basis. Although nearly always modeled as a population of isolated SMMs, the relative simplicity of their Ising-like moment and symmetry-restricted intermolecular dipole–dipole interaction offers a unique opportunity to scrutinize them for evidence of the effects of local correlation. In this work, we approach this challenge through the examination of three different crystal lattice configurations of the same anionic single-ion SMM [ErCOT_2_] by means of field-, temperature-, and time-dependent magnetometry and temperature-dependent X-ray crystallography. New quantitative insight is garnered from field-dependent magnetization using a fitting technique with parameters that are interpreted in terms of localized and intermolecular contributions induced by the dipole–dipole interactions relevant to the molecular crystalline arrangement. Additionally, this work characterizes the nature and consequences of a metamagnetic spin-flip transition that marks the separation between the correlated ground state that maximally internalizes local fields and the aligned excited state generated by external applied fields. By delving into the connections between single-molecule magnetism and solid-state magnetism, we seek to uncover the promising avenues for harnessing molecular building blocks to engineer materials with tailored magnetic properties that take advantage of spatial dimensionality.

## Experimental and Crystallographic Details

**Figure 1:**
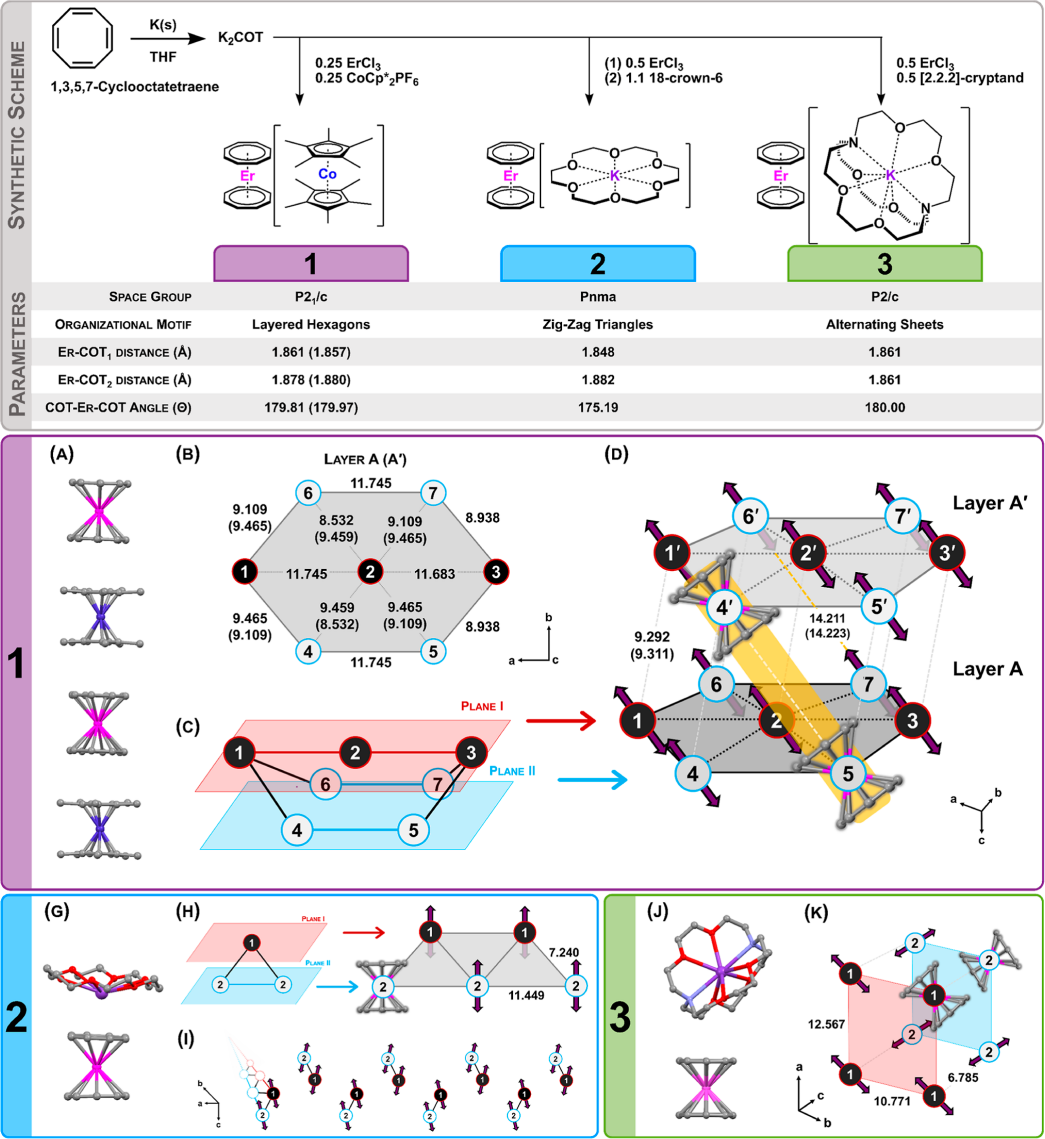
Synthetic scheme, crystallographic parameters, solid-state structures of unit cells, and organizational lattice motifs of **1**, **2**, and **3**. **(top)** Synthetic scheme of compounds **1**, **2**, and **3**. Below each compound are crystallographic parameters depicting the space group, organizational motif, Er-COT distances, and Er-COT-Er angles. Values in parentheses for crystallographic parameters represent those arising from the second unit in the unit cell. **(Box 1)** Crystallographic unit cell and arrangement of **1**. (A) Unit cell of **1**. Spheres in unit cells represent erbium (pink), cobalt (blue), and carbon (gray); hydrogen atoms have been omitted for clarity. (B) Structural parameters of two hexagonal motif layers within **1**; distances listed are in angstroms, with layer A′ shown in parentheses. Black and white circles represent magnetic units, corresponding to planes depicted in figure C (red, plane I; blue, plane II). (C) Exaggerated buckled-hexagonal motif of **1**. Parallelograms depict planes I (red) and II (blue), upon which lie magnetic units 1–3 (black circles, red outline) and 4–7 (white circles, blue outline), respectively. (D) Two stacked layers of the hexagonal organizational motif of **1**, demonstrating interlayer stacking interactions, highlighted in yellow. Distances shown are in angstroms. Two-sided arrows demonstrate the approximate anisotropy axes of magnetic units. **(Box 2)** Crystallographic unit cell and arrangement of **2**. (G) Unit cell of **2**. Spheres in unit cells represent erbium (pink), carbon (gray), oxygen (red), and potassium (purple); hydrogen atoms have been omitted for clarity. (H) Zig-zag triangular organizational motif of **2** and depiction of planes (parallelograms, red and blue) upon which lie magnetic units. Distances are given in angstroms. Black and white circles represent magnetic units, corresponding to the planes depicted in the figure. Two-sided arrows demonstrate the approximate anisotropy axes of magnetic units. (I) Lattice organization of **2**, demonstrating canting between triangular zigzag motifs within the crystal. Black and white circles depict magnetic units, as in figure H. **(Box 3)** Crystallographic unit cell and arrangement of **3**. (J) Unit cell of **3**. Spheres in unit cells represent erbium (pink), carbon (gray), oxygen (red), potassium (purple), and nitrogen (light blue); hydrogen atoms have been omitted for clarity. (K) Alternating sheets organizational motif of **3**, with black and white spheres depicting magnetic units on different planes. Two-sided arrows demonstrate the approximate anisotropy axes of magnetic units. Distances listed are in angstroms.

A series of three magnetic molecules were synthesized in which the magnetic unit is held constant while the charge-balancing cation is varied. The lanthanocene magnetic unit [ErCOT_2_]^−^ (bis(cyclooctatetraenyl)-erbium) was chosen due to its well-studied^10,27,30−38^ crystal field structure, highly axial ground state anisotropy, and well-defined SMM behavior. The axial single-ion anisotropy provides a nearly ideal magnetic building unit for the construction of anisotropic Ising-type^39^ lattices. Furthermore, the driving factor determining the single-ion anisotropy is the local crystal field environment, meaning that the molecular anisotropy axis, well-approximated by the structural COT-Er-COT vector, can be treated as a local Ising spin axis, reliably tethering the spin space to a concrete Cartesian direction. Charge-balancing cations chosen for this study were selected to drive differences in the crystal packing and orientations of magnetic units with respect to one another within the crystal lattice. As such, we synthesized near-linearly stacked bis(pentamethylcyclopentadienyl)-cobalt(III) bis(cyclooctatetraenyl)-erbium(III) (**1**, [CoCp*_2_][ErCOT_2_]), previously studied K(18-crown-6) bis(cyclooctatetraenyl)-erbium(III) (**2**, [K(18-C-6)][ErCOT_2_]),^37^ and near-orthogonal K([2.2.2]cryptand) bis(cyclooctatetraenyl)-erbium(III) (**3**, [K(crypt)][ErCOT_2_]; Figure 1, synthetic scheme). As the scope of this work was to analyze these compounds under the lens of dipolar coupling, it was pertinent to conduct a dilution study with a diamagnetic molecular analogue containing Y(III) to disrupt the crystalline dipolar network via random insertion of diamagnetic sites. Dilution of the parent erbium(III) compound to form **1-Y**, **2-Y**, and **3-Y** was achieved with a diamagnetic yttrium(III) analog in 5:95 Er/Y molar ratios. Yttrium was chosen due to its similar ionic radii to erbium and its ability to generate diamagnetic isostructural analogues of the parent erbium compounds.

Single crystal X-ray diffraction data reveal that the [ErCOT_2_] anionic units are similar across the three compounds of interest, with Er-COT distances within 1.85–1.88 Å. The COT_1_-Er-COT_2_ angle for **2** shows a slightly greater canting than **1** and **3** due to K-18C6 coordination on one side of the [ErCOT_2_]^−^ unit (Figure 1, parameters). To ascertain the similarity of the magnetic states at the level of the crystal field interactions, CASSCF calculations were performed within the SINGLE_ANISO module of OpenMolcas^40,41^ for **1**–**3**, with and without charge-balancing cations. CASSCF calculations provide consistently anisotropic ground states (*g*_*z*_ = 17.99, *g*_*x*_ = *g*_*y*_ = 0.00; KD_0_ = ± 15/2, 100% pure; Δ*E*_KD1_ ∼ 160 cm^–^^1^; Table S4). From these calculations, we conclude that the [ErCOT_2_] anionic unit provides a consistent basis for studying varying intermolecular interactions in crystalline lattices. To further develop this concept, a thorough analysis of the spatial relationship between molecules and thus their local anisotropy axes was made via X-ray crystallographic studies.

Compound **1** crystallizes in *P*2_1_/*c*, with two crystallographically distinct [ErCOT_2_]^−^ units in the unit cell (Figure 1A–D). Two analogous buckled hexagonal motifs (layers A and A′ in Figure 1B) stack down the *c*-axis of the crystal lattice. The hexagonal motif is buckled down the center of a “boat” hexagonal conformation with [ErCOT_2_]^−^ units in positions 1–3 located on slightly elevated plane I (black circles, red outline), and [ErCOT_2_]^−^ units in positions 4–7 on slightly lowered plane II (white circles, blue outline, Figure 1C). These hexagonal motifs are linked side-on (positions 4 and 5 to 6 and 7) and head-on (positions 1 to 3) across the ab-plane and stacked down the crystallographic *c*-axis (Figure 1D). In applying our previous heuristic dipolar coupling approximation,^27^ the angles between the following units predict ferromagnetic coupling: 1:2, 2:3, 4:5, and 6:7 (center to center and edge to edge), with antiferromagnetic coupling predicted to arise from all other interactions: 1:4, 1:6, 2:4, 2:6, 2:5, 2:7, 3:5, and 3:7 (center to edge). Importantly, the lattice configuration of **1** obtains a near-colinear head-to-head interlayer stacking of the ErCOT_2_ units, spaced by the [CoCp*_2_]^+^ charge-balancing cation, generating a pseudo-1D linear wire throughout the crystal lattice. This stacking interaction occurs by plane: plane I′, layer A′ stacks to plane I, layer A, positions 1′ to 2, 2′ to 3, and 3′ to 1. Correspondingly, positions 4–7 on plane II′ stack with plane II (4′:5, 5′:4, 6′:7, 7′:6; the 4′:5 stacking interaction is highlighted in yellow in Figure 1D). All interlayer interactions (head-on, as in 4′:5, and side-on, as in 4′:4) are predicted to be ferromagnetically coupled. This complex interleaving of molecular forms yields ferromagnetically coupled planes down the *c*-axis, complemented by intralayer antiferromagnetic interactions (*vide infra*). The simultaneous presence of hexagonal motifs and antiferromagnetic coupling is accompanied by the intraplanar buckling, which serves to alleviate magnetic spin-frustration on the triangular motifs within the hexagons. While certainly not the only force involved, the structure’s alignment to allow favorable Ising interactions indicates an unusually large level of structural stabilization based on the spin system.

Compound **2** crystallizes in *Pnma* with one crystallographically distinct [ErCOT_2_]^−^ unit within the unit cell, in two symmetry-related obtuse isosceles triangular “zig-zag” motifs traveling down the *b*-axis (Figure 1G–I). As in **1**, there are two planes upon which [ErCOT_2_] anionic units are positioned, one slightly elevated over the other. The anisotropy axes of the individual units are nearly parallel to one another, resulting in a near-90-degree angle of projection onto their internuclear axis (Figure 1H,I). Based on the mutual dipolar interaction of their large angular momenta, this spatial arrangement results in a minimization of the antiferromagnetic coupling energy. The two crystallographically generated zigzag motifs created by this interaction are canted with respect to one another (Figure 1I) and traverse the *b*-axis.

Compound **3** crystallizes in *P*2/*c*, with sheets of [ErCOT_2_]^−^ anionic units oriented in one direction across the ab-plane (Figure 1J,K). The sheets traverse down the *c*-axis, alternating between two nearly orthogonal orientations of the [ErCOT_2_] anionic units.

Intrigued by the relationship between the anisotropy axes and the crystallographic arrangement, we completed a temperature-dependent crystallographic analysis on **1** to garner insight into the important modes of structural relaxation and look for correlations to our magnetostructural parametrization (*vide supra*). In brief, a crystal of approximately 0.03 mm × 0.05 mm × 0.06 mm was identified and mounted on a Bruker ApexII-Ultra CCD with a microfocus rotating anode using a Mo(Kα) radiation source. Full collections were completed at seven temperatures from 200 to 80 K, and all structures were solved using direct methods via the SHELX routine and refined with SHELXL.^42^ At 200 K, the structure of **1** was solved in space group *P*2_1_/*m* with one crystallographically distinct [CoCp*_2_][ErCOT_2_] unit in the unit cell. There is only one hexagonal motif with added symmetry generated by the mirror plane. At 80 K, the structure was solved into *P*2_1_/*c* with the two analogous hexagonal motifs, as previously discussed. The structural changes of the hexagonal motifs were tracked by comparing the 200 K, *P*2_1_/*m* (hot) and 80 K, *P*2_1_/*c* (cold) structures (Figure S4A–C, green: contractions; purple, expansions). The structural expansions upon cooling accompany relaxation of the spin frustration within the lattice while also resulting in an increase in the distance between ferromagnetically coupled sheets. In layer A, we see expansion on one side of the hexagon away from the central positions. Conversely, in layer A′, the expansion occurs on the other side of the hexagon. In looking at the lattice (Figure S4A–C), we see a general expansion outward from the center positions, balanced by a contraction along the lengths of the hexagons and a contraction in the stacking between layers A and A′ (Figure S4A). Although it is not possible from the current evidence to say definitively that the symmetry lowering is the result of “freezing out” a spin-phonon coupling, it is consistent with both the structural evidence and the small energies organizing the molecular lattice. These temperature-dependent structural changes serve as clues toward building a more complex and nuanced model of their behavior in the context of their environment. The SMM model is effective at explaining a broad range of magnetic behavior from the perspective of an isolated spin system, but the strong and often highly directional properties of SMMs can have interesting effects on the relatively flat energy landscape of the molecular crystalline environment that warrant a closer study. Observing the range of closely related organizational motifs, their interconversion as a function of temperature, and their telling relationship with the anisotropy axes, we turned to a full characterization of the magnetic behavioral differences between **1**, **2**, and **3**.

## Results and Discussion

**Figure 2:**
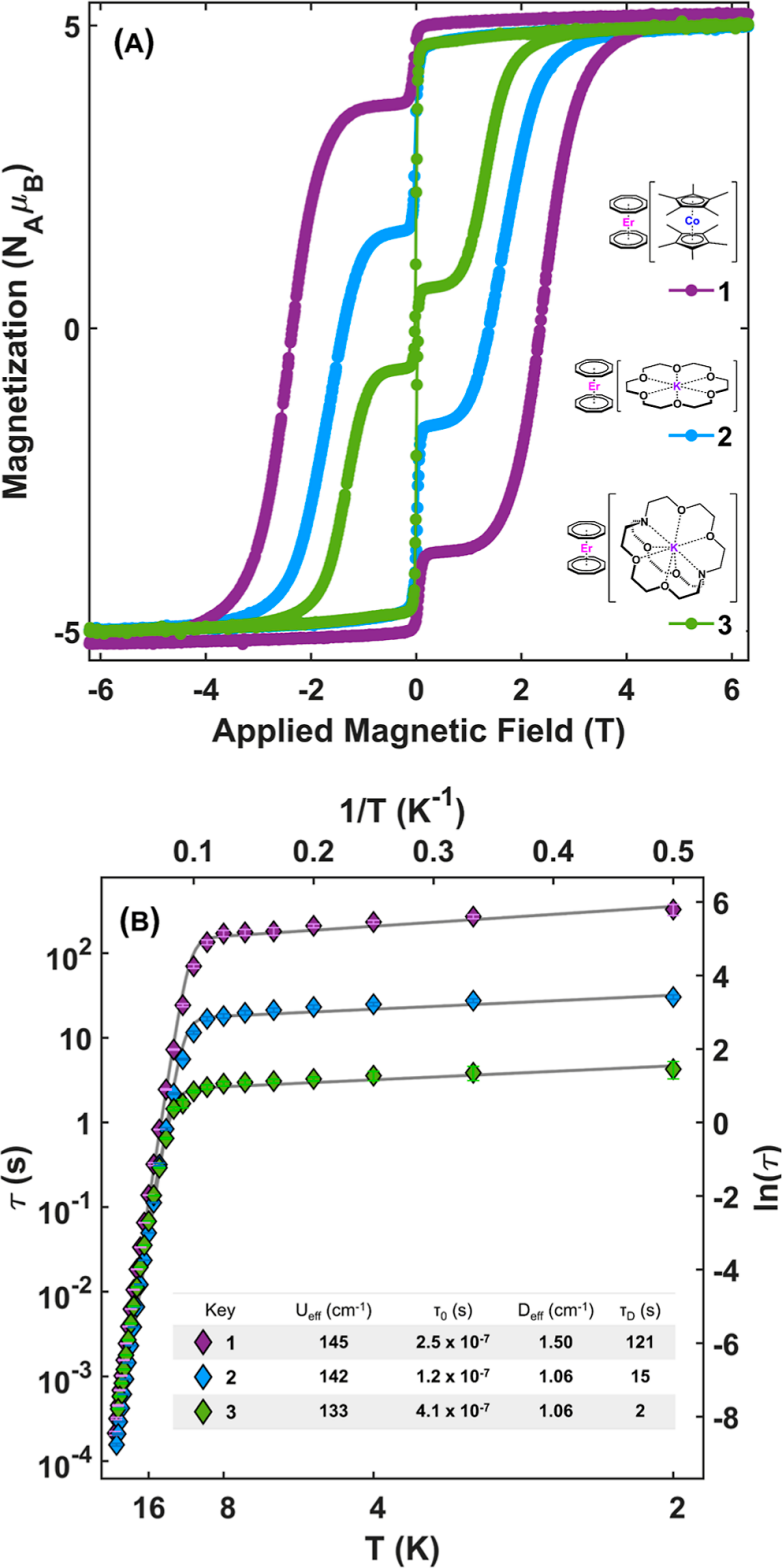
(A) Isothermal magnetization of **1**, **2**, and **3** (purple, blue, and green circles, respectively; lines are guides for the eye). Data were collected at *T* = 2 K at a 60 Oe s^–1^ magnetic field sweep rate. (B) Arrhenius plots of relaxation times versus temperature for **1**, **2**, and **3** (purple, blue, and green diamonds, respectively). Error bars within diamond markers demonstrate the upper and lower error limits of the τ values. Gray lines are fits to a multiterm relaxation model. The inset table shows fit parameters.

To investigate the effects of crystal lattice organization on the magnetic properties of our compounds, we collected static magnetic isothermal magnetization data and dynamic magnetic data via standard magnetometry techniques. Representative isothermal sweeps of magnetization versus magnetic field are shown in Figure 2A, collected at *T* = 2 K. All compounds saturate near 5 μ_B_. Compounds **1** and **2** both display open hysteresis (60 Oe s^–1^), with coercive fields of *H*_c_ = 2.4 and 1.7 T, respectively. Compound **3** displays a waist-restricted hysteresis loop with no evidence of coercive field. Arrhenius relaxation plots of τ versus temperature and corresponding fits of **1**, **2**, and **3** are shown in Figure 2B. As observed previously,^27^ the low temperature relaxation regime is characterized by Arrhenius-linear processes characterized by weak temperature dependence and highly impeded transition rates. The phenomenological model for this process uses τ_D_ to describe the attempt time and *D*_eff_ to describe the barrier in the low temperature regime (analogous to the high-temperature variants, τ_0_ and *U*_eff_). Interestingly, this fitting model is effective despite the difference in magnetic structure compared to other systems where it has been found effective. When employed previously in the study of locally dipole–dipole coupled molecules, both τ_D_ and *D*_eff_ corresponded well to the expected behavior induced by the coupling of local, highly anisotropic Ising states. In the current system, however, the interpretation is far more complex as the Ising dipoles couple in three dimensions. The fitting of the experimental data, followed by the extraction of these parameters, allows us to see the stark differences in relaxation dynamics in the cold-temperature regime below 10 K, corresponding well to the differences seen in isothermal magnetization data. Experimental data falling within the Orbach regime (high temperature) are consistent between compounds and correspond well to the CASSCF calculated energy splitting between the ground state Kramers doublet (KD_0_) and the first excited state Kramers doublet (KD_1,_ 160 cm^–^^1^, Table S4). However, within the low-temperature regime, we see a three-order of magnitude difference in dipolar attempt time, τ_D,_ at 2 K, varying between 121, 15, and 2 s for **1**, **2**, and **3**, respectively (Figure 2B, inset). A work published recently notes a similar relationship in the low-temperature regime on a series of modified [ErCOT_2_]^−^ substituted compounds,^38^ attributing the differences to a dipolar interaction.

**Figure 3:**
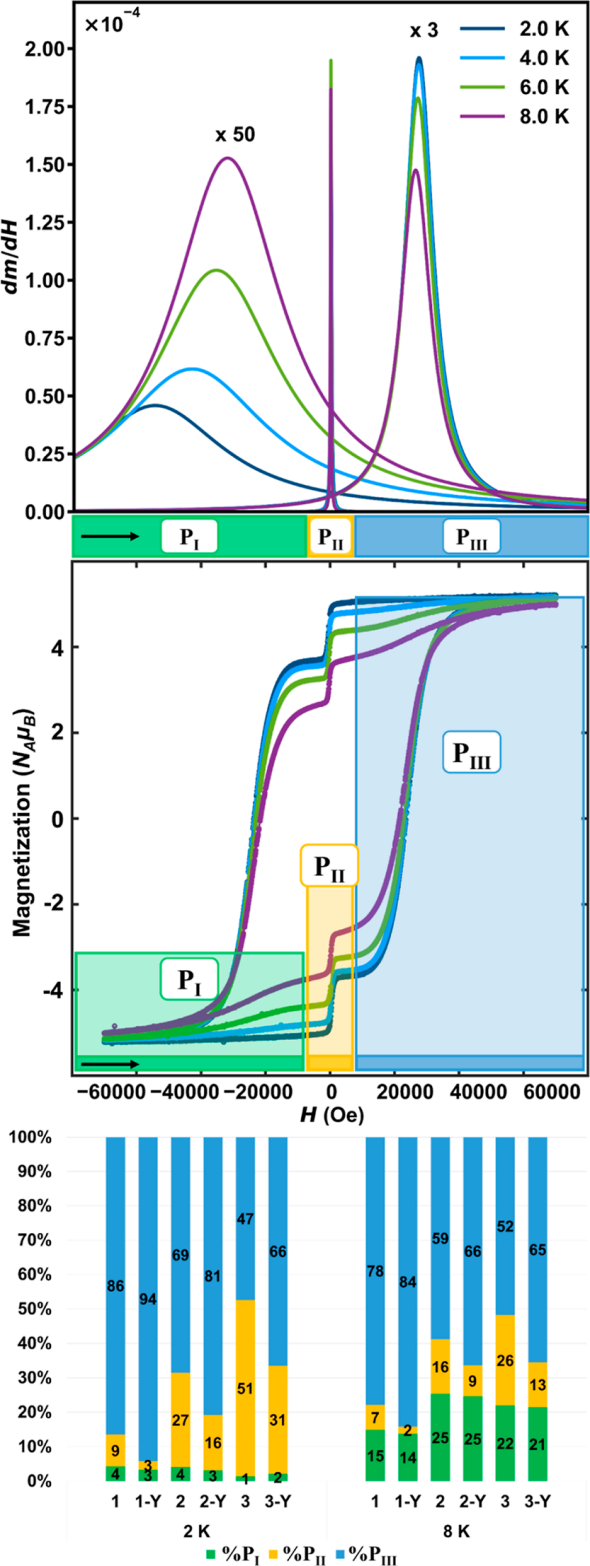
Modified Cauchy fits (top) to reverse sweep of isothermal magnetization loops (middle) collected at *T* = 2, 4, 6, and 8 K for **1** at a constant sweep rate of 60 Oe s^–^^1^. **P**_**I**_ (green), **P**_**II**_ (yellow), and **P**_**III**_ (blue) correspond to processes (peaks) observed from fitting the data. **P**_**I**_ and **P**_**III**_ are magnified by 50 and 3 times, respectively. The black arrow designates the direction of the sweeping field. (bottom) Percent contribution of **P**_**I**_ (green), **P**_**II**_ (yellow), and **P**_**III**_ (blue) for compounds **1**, **2**, and **3**, and their diluted analogues, **1-Y**, **2-Y**, and **3-Y** from modified Cauchy distribution fits of magnetization isotherms at 2 and 8 K.

Upon seeing these initial differences in magnetic behavior, we were curious to quantify them further. As such, we collected isothermal magnetization hysteresis loops at *T* = 2, 4, 6, and 8 K for **1**, **2**, and **3** and their diluted analogues, **1-Y**, **2-Y**, and **3-Y**, respectively, and fit them using a Cauchy statistical distribution model (Figure 3). The Cauchy distribution can be used to model a wide variety of statistical behaviors arising from quantum mechanical systems, and specifically, the Cauchy probability density function (PDF) yields the Lorentzian peak shape consistent with a homogeneous distribution. We have recently reported on the use of this statistical distribution with regards to magnetization quantification in nanoparticles^43^ and extended it to molecular systems in this work. Utilizing *multi_Cauchy*,^44^ an open-source software package, data were fit to a combination of three unique Cauchy cumulative distribution functions (CDF). To better illustrate the temperature dependence and subtle variations in the peaks, the fit parameters were applied to analytical forms of the PDF formulation of the Cauchy distribution and plotted. This technique allows us to quantify, track, and compare (de)magnetization processes occurring across all compounds in a model-agnostic manner. We are able to extract and quantify parameters from the fits, such as **H**_**P**_ (the field at which a transition takes place), **γ** (broadness of the peak; half width, half max), and percent contribution of the process (*P*_*n*_/∑*P*_I_ + *P*_II_ + *P*_III_). All compounds, including their diluted counterparts, show the presence of three (de)magnetization events: **P**_**I**_, **P**_**II**_, and **P**_**III**_. Figure 3 shows fits to the reverse sweeps of magnetization isotherms of **1** in which these three (de)magnetization processes are evident: **P**_**I**_ (green, broad, ∼ −40 kOe), **P**_**II**_ (yellow, sharp, ∼ 0 Oe), and **P**_**III**_ (blue, broad, ∼ 24 kOe). By quantifying the sample magnetization in terms of its change with respect to the field, we can more clearly delineate the importance of the spin, lattice, and bath as the system approaches and is at equilibrium. As temperature is increased from 2 to 8 K, **P**_**I**_ increases in percent contribution and decreases in magnitude of **H**_**P**_. **P**_**II**_ and **P**_**III**_ both decrease in percent contribution, and H_PIII_ magnitude decreases as well. The trend in temperature dependence follows for all compounds, concentrated and dilute, except for **3**, **P**_**III**_, which increases in percent contribution and **H**_**P**_ with increasing temperature (Figure 3; ESI, Section S7). Curiously, the percent contribution of the weakest contribution, **P**_**I**_, appears to be nearly unchanged regardless of dilution. Quantification of **P** gives us a more concrete, albeit empirical, way to discuss time, temperature, and field dependence within the blocked regime where a model generated from noninteracting SMMs may fail. The most commonly discussed manifestation of this failure is the zero-field avalanche effect observed when the momentum of dipole flips can induce the flips of nearby spins in a self-propagating manner that drastically accelerates the approach to equilibrium of the entire sample. Modeling or predicting this effect or any other inter-SMM relaxation dynamics (e.g., spin glass formation, spin clustering dynamics, or manifestations of magnetic order) is complex, and the behavior often goes unnoticed or its effects are conflated with the slow-relaxation dynamics of an SMM. With this technique, we are able to quantify **P**_**II**_, the drop(rise) near zero field that is typically attributed to QTM processes or magnetic avalanche^37,45,46^ and see that the diluted counterparts show decreased percent contribution in **P**_**II**_, as seen in many past works,^37,47−49^ with increased sharpness, as evidenced by the low **γ** parameters (e.g., **P**_**II**_ at 2 K: γ_**1**_ = 195, 9.2% contribution versus **γ**_**1-Y**_ = 9, 2.6% contribution, Tables S5 and S6).

**Figure 4:**
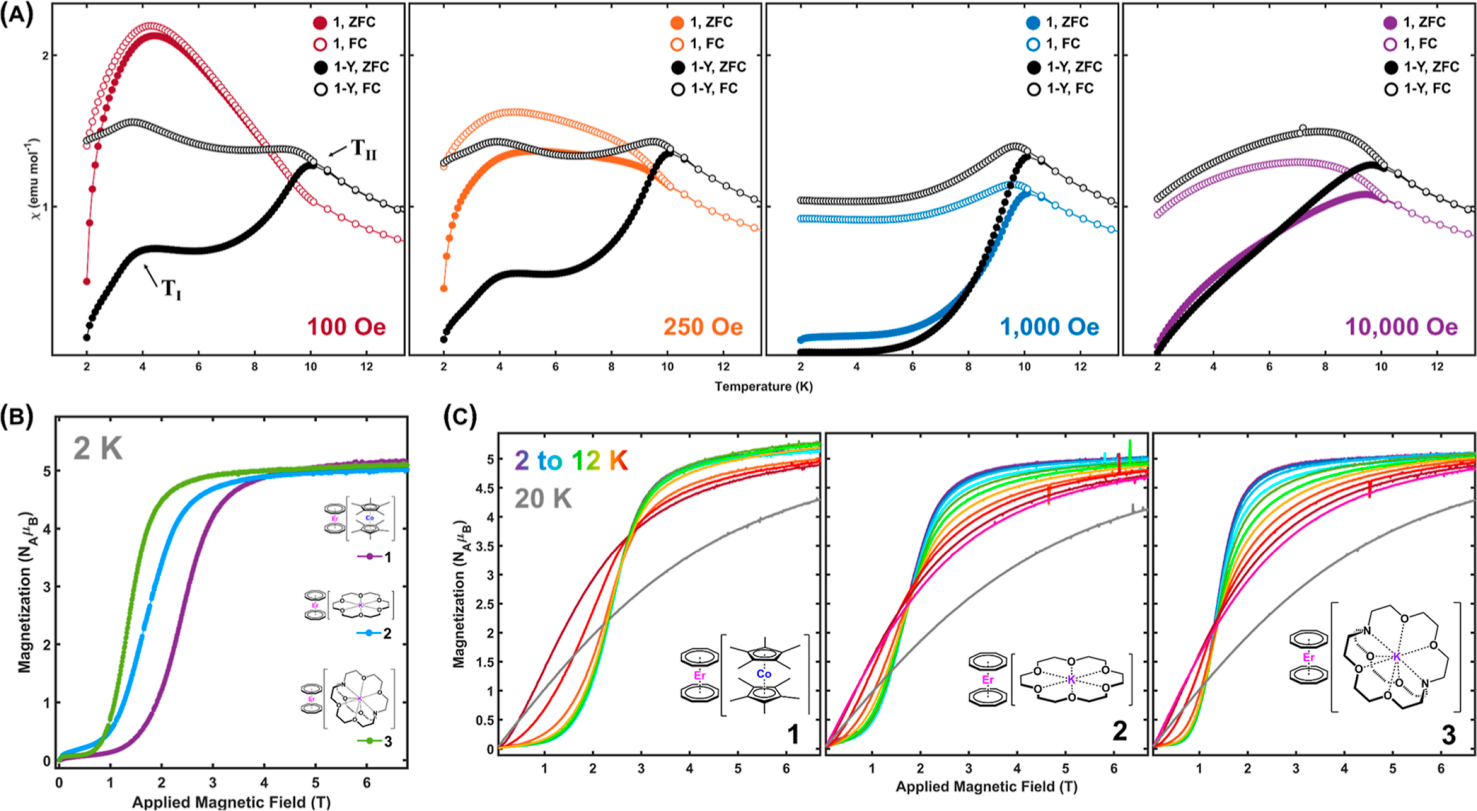
(A) ZFC (filled markers) and FC (open markers) susceptibility data for **1** (colored circles) and **1-Y** (black and white circles) under varied applied fields. **T**_**I**_ and **T**_**II**_ are susceptibility transition event temperatures, as discussed in the text. Lines are guides for the eye. (B) Isothermal virgin magnetization curves collected at applied fields of H = 0–7 T at 2 K for **1** (purple), **2** (blue), and **3** (green) at a constant sweep rate of 60 Oe s^–^^1^ using vibrating sample magnetometry VSM. (C) Isothermal virgin magnetization curves collected at applied fields of *H* = 0–7 T at *T* = 2–12 (purple to red) and 20 K (gray) for **1**, **2**, and **3** at a constant sweep rate of 60 Oe s^–^^1^ in VSM mode. Note that the low-temperature curves overlap in **1**.

Furthermore, when we subjected our samples to an applied field for ZFC and FC susceptibility measurements, we discovered that many of the complex and often undiscussed variations in behavior observed for SMMs could be correlated to susceptibility processes defined in terms of **P**_**I–III**_ (Figures 4A and S5–11, S17–22, S28–33). The overlay of concentrated **1** and dilute **1-Y** susceptibility plots shows a combination of expected and unexpected behavior for an SMM (Figure 4A, colored and black and white circles, respectively). The data collected at a field of 1000 Oe is indicative of standard SMM behavior. A low-temperature “blocked” region exists under zero-field-cooled (ZFC) conditions where the SMM is unable to respond to the field. The sample is unable to establish the new equilibrium condition, as determined by the applied field, until the temperature is raised enough to overcome the barrier to magnetic relaxation. When the system is cooled under an applied field (field-cooled, FC), the magnetization immediately responds because the equilibrium condition was in place as the system cooled. The temperature marked **T**_**II**_ is consistent with the expected convergence for the ZFC/FC behavior of **1**, given its relaxation dynamics. Deviations from this behavior at both higher and lower fields are drastic and indicative of more complex behavior involved outside the 1000 Oe measurement. The important information about deviations from ideal SMM behavior can be summarized as follows: for all samples in all magnetic fields, the diluted sample conforms to the SMM behavior better than the corresponding concentrated sample. The deviation from the ideal behavior occurs at high and very low applied magnetic fields. The bifurcation between ZFC and FC susceptibility curves for **1** is minimal at low field, leading to a broad, highly susceptible feature, **T**_**I**_ ∼ 4 K. With increasing field toward 1000 Oe, eventually only a small shoulder remains at **T**_**I**_. This surprising, dramatically field-dependent behavior yielding two maxima in magnetic susceptibility is seen consistently in all our samples, with similar trends in behavior between concentrated and diluted species (Figures S5–S11, S17–S22, S28–S33). These trends suggested that **1**–**3** could all display collective spin interactions of varying magnitudes. This behavior is perhaps most striking in **1**, where a net cooperative interaction results in a large and broad enhancement of susceptibility over the entire low temperature range. This interaction can be suppressed by a relatively weak applied field at all but the lowest temperatures. It can also be weakened by generating defects in the dipolar lattice through magnetic dilution.

It is worth noting that additional complexity in the coupling and relaxation behavior of SMMs arising from their crystalline arrangement may be far more common than reported, especially for single-ion systems in which axial anisotropy plays such a key role. Revealing this behavior, however, requires nonstandard measurements and analysis for SMMs, such as the study of the demagnetization curvature, virgin curve analysis, and low-field susceptibility measurements. The precedence for long-range coupling of highly anisotropic spin centers has a rich history of study in the solid-state physics magnetism community, where the presence of multiple susceptibility peaks is used to detect potential spin-flop, spin-flip, or metamagnetic phase transitions.^50−59^ Given the many intrinsic similarities between molecular crystals of lanthanide single-ion SMMs and solid-state anisotropy-driven magnetic materials, the isothermal virgin magnetization curves from *H* = 0–7 T at *T* = 2–12, 20 K (Figures 4B,C and S16, S24, and S35) were collected. These data, in connection with the high degree of anisotropy of the [ErCOT_2_] anionic dipolar spin network, confirm the presence of a metamagnetic spin-flip transition in all of our compounds below their 10 K blocking temperature. This is especially evident in the lower temperature curves (Figure 4B, 2 K data), where we maintain a minimal magnetization of the AFM-coupled ground state, followed by an abrupt magnetization event with a small change in applied field. The spin-flip transition takes place at 2.4, 1.7, and 1.3 T for **1**, **2**, and **3**, respectively, and brings all samples to their collective fully magnetized FM-coupled state. Interestingly, this metamagnetic spin-flip transition corresponds with the field at which **P**_**III**_ occurs (H_PIII_) at 2 K. The spin-flip transition is observed in **1**–**3**, yet each species displays aspects unique to its magnetostructural arrangement. In **1**, the overlap of curves between 2 and 8 K show that the metamagnetic spin-flip transition is nearly temperature-independent with a sudden change in magnetization curvature, whereas both **2** and **3** shows a far smoother transition over the entire temperature range.

Let us summarize the findings of this work prior to analysis and comparison between the molecular properties preserved versus modified in the solid state. The following are consistent across all compounds and can be attributed to inherent characteristics of the [ErCOT_2_]^−^ anisotropic unit: magnetization saturation (M_sat_ ∼ 5 μ_B_), the presence of a near-zero-field drop in magnetization (**P**_**II**_), high-temperature relaxation dynamics driven by an Orbach barrier originating from the local crystal field (∼160 cm^–1^) and a spin-flip transition resulting from competition between the intermolecular magnetic dipole network and the applied magnetic field. Characteristics that differ across the three compounds and thus should be discussed as effects modified by solid-state arrangement are the coercive field (*H*_c_), the intensity of the near-zero-field magnetization drop (percent contribution of **P**_**II**_), the low-temperature relaxation dynamics (below 10 K), the field-dependency of the spin-flip transition, and the temperatures of the phase transitions.

The high-temperature relaxation dynamics are consistent across all compounds due to the same Orbach barrier imposed by the [ErCOT_2_]^−^ unit. This has been discussed thoroughly in the SMM literature and will not be discussed in this work.^6−8,60^ The saturation magnetization is consistent across all of our compounds, which again signifies that this feature can be attributed to preserved single-ion behavior. The cascading event near zero field, **P**_**II**_ (Figures 3, S15, S23, and S34), is present in all compounds, and its percent contribution toward demagnetization is decreased in the diluted counterparts. This also has precedence in the literature and can be attributed to self-propagating relaxation in the QTM regime falling under the category of magnetic avalanche effects.^37,45−49^ In keeping with theory and previous observations, the relative percentage of the sample that relaxes via QTM (**P**_**II**_) can be minimized through magnetic dilution. In dilute spin systems, local dipole fluctuations are limited, which inhibits further nucleation and growth of spin clusters. The spin-flip transition associated with metamagnetism is often described in solid state systems such as DyPO_4_, FeCl_2_, and others,^61−64^ as well as heavy Fermion systems^65−68^ occurs in electronic structures where an AFM ground state responds to a small change in applied field to yield a dramatic increase in magnetization to become a spin-polarized FM-type state. The characteristics necessary for metamagnetic spin-flip transitions are strong anisotropy and competing interactions within sublattices.^63,69−71^ Compounds **1**–**3** are composed of highly anisotropic, Ising-type magnetic units, all of which have distinct crystallographic organizational motifs (Figure 1) defining the low-lying magnetic structure. In that light, the presence of a metamagnetic phase transition is entirely unsurprising and yet uncommon for single-molecular magnetic systems.

The collection of virgin isothermal magnetization curves offers an interesting corroboration of the spin-flip transition in all three of our compounds. This transition happens at applied fields of *H* = 2.4, 1.7, and 1.3 T for **1**–**3**, respectively, implying that the crystal lattice organizational motifs are responsible for the changes and that the 3D, ferro-, and antiferromagnetic stabilization proffered by **1** leads to the strongest resistance to the spin flip transition. By contrast, the nearly orthogonal arrangement dominating the lattice of **3** is more easily broken by the external field. This behavioral trend corresponds well to the transitions in magnetization we tracked from the full isothermal magnetization loops (Figure 3) and the coercive fields of the compounds and demonstrates that the starting ground state of all of our compounds is, as we predicted through crystallographic analysis, dominated by AFM-coupling. There is one stark difference across the series of compounds: the applied field necessary to enable the spin-flip transition. If a greater field is needed to propagate a transition, this speaks for the coupling strength in the ground state prior to any application of field. This means that the internal fields generated within the organizational structures of **1**, **2**, and **3** differ in their propensity for coupling, enabling the elongation of relaxation times we see in the Arrhenius plots (Figure 2B). This is likely evidenced by the alignment of the anisotropy vectors within the organizational motifs in the synthesized crystal domains. All anisotropy vectors of **1** point in the same direction, whereas the anisotropy vectors of **2** and **3** offer less synergistic organization. Saturation magnetization values demonstrate that these differences are overcome by a large applied magnetic field for each of the compounds. This implies that the nuances of magnetic behavior are driven by the initial ground state of the coupled crystalline system. While these data can shed further light on what is or is not a magnetic effect of truly magnetic origin, this question is somewhat specious, as the existence of the SMM distinct from its environment is both theoretically and practically tenuous. More importantly, a better understanding of the interplay between molecular and intermolecular effects expands the potential materials toolbox and application space open for potential synthetic control by molecular magnetochemists.

Now, let us further examine the curious features of the two susceptibility peaks in our data, coupled to the transitions seen in isothermal magnetization hysteresis loops. The presence of multiple susceptibility peaks and field-dependent susceptibility events have been discussed in the solid-state literature for CeGe,^50^ lanthanide-containing double perovskites,^52^ Gd/TbAuAl_4_Ge_2_,^53^ and others.^50,51,54−59^ Most works discuss these kinds of anomalous events as magnetic phase transitions with long-range ordering and the potential presence of spin rearrangement or polarizability. Consistently, these multiple peaks are assigned as Neel temperatures and attributed to phase transitions occurring within the compound. While the FC/ZFC susceptibility behavior of **1-Y** (Figure 4, **T**_**I**_ and **T**_**II**_) involves the local slow relaxation dynamics of the SMM, the presence of phase transition behavior is viable and, in some cases, a likely effect as well. The structural changes we tracked during our temperature-dependent crystallographic study (Figure 1) show that a symmetry decrease in the lattice can change the energy of the dipolar coupling. While all complexes and their diluted counterparts display the effects we study, the particulars vary widely, indicating that the nuances are characteristic of solid-state organizational structures within the materials and may change based on small differences in dilution ratios or in the formation of domain structures^72^ within the crystals. The concentrated analogues, **1–3**, see a significant broadening of the same susceptibility peaks, especially at lower applied fields. This implies that intermolecular or interlayer interactions between the magnetic unit decrease the resolution with which we can resolve these events by modulating the interaction of states biased by the applied field.

Finally, we hypothesize that the transitions we see in DC susceptibility, **T**_**I**_ and **T**_**II**_, are related to and can inform the processes we track in isothermal magnetization (**P**_**I**_, **P**_**II**_, and **P**_**III**_, Figure 3). **T**_**I**_ is strongly influenced by even small fields, similar to **P**_**II**_, the near-zero transition seen in isothermal magnetization loops. **T**_**I**_ becomes a probe into low-field nuances around **P**_**II**_, showing how the long-range stabilizing effect of the dipolar lattice homogenizes the internal field and restricts the rate of QTM.

## Conclusions

We have presented a series of three ErCOT_2_ compounds and their diluted analogues in which we have held the magnetic unit constant and modified the organizational motifs of the crystal lattice through judicious choice of charge-balancing cation. These compounds vary dramatically in their low-temperature relaxation behavior, attributable to dipolar interactions functioning within the organizational motifs of the crystal lattice. In some cases, it is possible to differentiate between effects arising from inherent single-ion properties and those arising from collective interactions, but often the effects are interrelated, coupled, or synergistic in ways that make such distinctions counterproductive. We have shown the surprising presence of structural changes indicative of coupling between the lattice and spin and a freezing out of a symmetry-lowered mode, as evidenced by a temperature-dependent crystallographic analysis.

The collection of data in this work extends measurements common to molecular magnetochemists to characterize multidimensional magnetic interactions, a process that could yield a wealth of new insight from the already extensive catalog of high-anisotropy crystalline synthetic materials. Low-field magnetic analysis shows how insight into factors such as the impact of intermolecular interactions on spin-phonon coupling, magnetostructural rearrangements, and relaxation dynamics can be revealed. Additionally, the discussion of solid-state-like effects and behaviors on SMM systems is nearly absent from the literature and the field. To our knowledge, our work on the [ErCOT_2_]^−^ magnetic unit is the first to describe the effects of metamagnetism on a crystalline lattice of SMMs. The [ErCOT_2_] magnetic system serves as an ideal candidate for the study of anisotropic Ising systems due to the presence of the real-space tethering of the local anisotropy axes and their use in interpreting the complex interrelation of localized and delocalized effects occurring in magnetic molecular crystals. As a means of quantifying our discussion without narrowing its scope, we have extended our isothermal magnetization fitting technique (Cauchy CDF) to molecular-based systems to facilitate comparison between (de)magnetization processes in any system with nonlinear magnetization vs field. This provides a quantitative basis for the development of theory and comparison to existing models for SMM-based systems. We hope this work encourages practicing magnetochemists to analyze magnetic behaviors in varied applied fields and to dig deeper into solid-state-like attributes that present viable pathways to the development of molecular spin-based technologies.

## Methods

All magnetic data collection was completed on crushed microcrystalline samples layered with eicosane in custom quartz tubes sealed under a vacuum on an MPMS3 SQUID. Isothermal magnetization data were collected in VSM mode at varied temperatures at a magnetic field sweep rate of d*H*/d*t* = 60 Oe/s. To quantify the magnetization curves for comparative analysis, fits to a modified Cauchy distribution were carried out following a previously published protocol with the *multi_Cauchy* software package.^43,44^ Dynamic magnetic properties were probed via standard AC susceptibility techniques with an extended frequency space (1000–10^–5^ Hz) analyzed by a previously described waveform technique.^27,33^ AC susceptibility and waveform data were fit to a Debye model, and the corresponding relaxation data were fit to a multiterm relaxation equation including an Orbach and dipolar relaxation term (eq S1). Temperature-dependent magnetic susceptibility data were collected as ZFC and field-cooled (FC) susceptibility data in the DC mode at biasing fields of *H* = 100, 250, 500, 750, 1,000, 10,000, and 40,000 Oe. ZFC data were collected by first cooling the sample to *T* = 2 K without an applied biasing field, then applying the biasing field of choice to the cooled sample and collecting data as the temperature was incremented up to *T* = 300 K. FC data were collected in sequence, with the application of the external biasing field of choice during the initial cooling step. Virgin magnetization curves were collected in VSM mode at varied temperatures at a magnetic field sweep rate of d*H*/d*t* = 60 Oe/s. Details of the crystallographic temperature study, synthetic methodologies relating to the dilution studies, and computational and fitting methods are discussed further in the Supporting Information.

## Supporting Information Available

Preparative details, sample characterization, and physical and computational details for all compounds (PDF)
