# Supplementary material for: Molecular Network Approach to Anisotropic Ising Lattices: Parsing Magnetization Dynamics in Er3+ Systems with 0–3-Dimensional Spin Interactivity
Source: J Am Chem Soc. 2023 Sep 29;145(40):22265–75. doi: 10.1021/jacs.3c08946 (PMC10571078; doi:10.1021/jacs.3c08946)
Supplement: Supplementary file 1 — ja3c08946_si_001.pdf [file ja3c08946_si_001.pdf]

Electronic Supplementary Information

A molecular network approach to anisotropic Ising lattices: Parsing magnetization dynamics in Er^{3+} systems with 0 – 3 dimensional spin interactivity

Angelica P. Orlova, Maxwell S. Varley, Maximilian G. Bernbeck, Kyle M. Kirkpatrick, Philip C. Bunting, Milan Gembicky, Jeffrey D. Rinehart*

Department of Chemistry and Biochemistry, University of California, San Diego,
*La Jolla, California 92093, United States. *E-mail:* jrinehart@ucsd.edu

CONTENTS

1 Designations of Work 3

2 Synthetic & Experimental Details 4

2.1 General Considerations 4

2.2 Dipotassium cyclooctatetraenide, K_2COT 4

2.3 Potassium bis(η^8-cyclooctatetraenyl)erbium(III), $K[Er(COT)_2]$ 4

2.4 Compound **1**, $[CoCp^*_2][Er(COT)_2]$ 4

2.5 Compound **2**, $[K\text{-}(18\text{-crown-}6)][Er(COT)_2]$ 5

2.6 Compound **3**, $K([2.2.2]cryptand)[Er(COT)_2]$ 5

2.7 Details of Dilution Study 5

3 Crystallographic Details 6

4 Magnetometry Details 9

4.1 Magnetic Data of Compounds **1** & **1-Y** 10

4.2 Magnetic Data of Compounds **2** & **2-Y** 17

4.3 Magnetic Data of Compounds **3** & **3-Y** 24

5 FT-IR Spectra 31

6 Computational Details 33

7 Curve Fitting and Analysis 34

8 References 50

1 DESIGNATIONS OF WORK

APO wrote the manuscript with input from all authors, synthesized the compounds, completed all characterization, computational calculations, and analysis. MSV assisted APO in synthesis, characterization, and computational calculations. MGB offered insight and assistance with manuscript preparation, characterization, and synthesis. KMK completed all Cauchy fitting analysis using code written by PCB. MG provided crystallographic assistance for compounds in this work and in the crystallographic temperature study. JDR oversaw the project and assisted with writing the manuscript.

2 SYNTHETIC & EXPERIMENTAL DETAILS

2.1 General Considerations

All manipulations were conducted under anaerobic, anhydrous conditions under an atmosphere of N_2 in a Vacuum Technology Inc. glovebox. All glassware was dried at 160 °C overnight prior to use. Tetrahydrofuran (THF) and pentane were dried on an activated alumina column and stored over a 1:1 mixture of 3 and 4 Å molecular sieves for at least two days before use. Erbium trichloride (Alfa Aesar), yttrium trichloride (Alfa Aesar), 1,3,5,7-cyclooctatetraene (Acros Organics), bis(η^5-pentamethylcyclopentadienyl)cobalt(II) hexafluorophosphate ([CoCp*$_2$][PF_6]) (Sigma Aldrich, Strem), 1,4,7,10,13,16-hexaoxacyclooctadecane (18-crown-6) (Alfa Aesar), and 4,7,13,16,21,24-hexaoxa-1,10-diazabicyclo[8.8.8]hexacosane ([2.2.2]cryptand) (VWR) were all used as received. CHN elemental analyses were performed by Midwest Microlab, Indianapolis, IN, U.S.A.

2.2 Dipotassium cyclooctatetraenide, K_2COT

An excess of potassium metal was washed several times with THF until the resulting solution was clear with a slight blue tint. To the clean potassium was added ca. 15 mL THF and the container was placed in the freezer (-30°C) to cool. Meanwhile, 1 mL COT was diluted in ca. 5 mL THF. This solution was also placed in the freezer to cool. After about 30 minutes, a slow, dropwise addition of the COT solution to the potassium in THF was performed. The color of the reaction mixture was observed to change from amber-yellow to dark brown. The reaction mixture was left in the freezer for 24 hours, after which time the dark brown solution was collected and centrifuged. The clear, dark brown supernatant was collected and concentrated *in vacuo*. This solution was returned to the freezer and large, light brown crystals grew within 24 hours. These crystals were dried *in vacuo* and washed with pentane. After crushing, the off-white micro-crystalline powder was used in further reactions.

2.3 Potassium bis(η^8-cyclooctatetraenyl)erbium(III), K[Er(COT)$_2$]

Synthesis was adapted from previous methods.[1] K_2COT (2 eq., 0.4267 g) was dissolved in ca. 8 mL THF and cooled in a -30°C freezer. In a separate vial, $ErCl_3$ (1 eq., 0.3202 g) was placed in ca. 2-3 mL THF and stirred, resulting in a pink slurry. The cold K_2COT was added slowly, dropwise to the stirring $ErCl_3$ slurry, resulting in a cloudy, yellow mixture. This mixture was left to react for 24-48 hours at -30°C, at which point it was centrifuged yielding a clear yellow supernatant and yellow pellet. The supernatant was collected, and the thick yellow pellet was extracted three times with ca. 5 mL THF. All fractions were combined into one vial and solvent was removed *in vacuo*, yielding a yellow microcrystalline powder. This was washed with several portions of pentane, dried, and used in downstream synthesis (0.4080 g, Yield: 84 %).

2.4 Compound **1**, [CoCp*$_2$][Er(COT)$_2$]

To a stirring suspension of $ErCl_3$ (0.1052 g, 0.3845 mmol) and [CoCp*$_2$][PF_6] (0.1815 g, 0.3826 mmol) in ca. 4 mL THF was added dropwise a cooled solution of K_2COT (0.2788 g, 1.529 mmol, ca. 8 mL THF). The reaction mixture immediately took on a dark-brown color and was allowed to stir at room temperature for 24 hours. After this period, insoluble impurities were removed *via* centrifugation, followed by filtration through a glass filter. The clear, brown supernatant was concentrated *in vacuo* and crystallized in the freezer (-30 °C). After 48 hours, small, violet crystals of X-ray diffraction quality had grown (0.0692 g, Yield: 25.6%). CHN analysis (calculated, found) for [$ErCoC_{36}H_{46}$]: C (61.34, 60.68); H (6.58, 6.65); N (0.00, 0.00).

2.5 Compound **2**, [K-(18-crown-6)][Er(COT)$_2$]

Synthesis was adapted from previous reported methods.[1] 1.1 equivalents of 18-crown-6 (0.1242 g) and 1 equivalent of K[Er(COT)$_2$] (0.1773 g) were dissolved separately in ca. 5 mL of THF each and cooled in a -30 °C freezer. The 18-crown-6 solution was then added to the K[Er(COT)$_2$] solution, dropwise with stirring. The yellow solution gradually became cloudier over the course of 5 min and was allowed to react for 24-48 hours. The reaction mixture was then centrifuged, resulting in a clear yellow supernatant and yellow pellet. The supernatant was concentrated *in vacuo* and then left undisturbed at room temperature for ~3 hours, at which point crystals began to form and the solution was transferred to a -30°C freezer to continue crystallization. The pellet was resuspended in THF and stirred at 48°C for 24 hours. The resulting mixture was centrifuged and concentrated following the aforementioned procedure and moved to the freezer to crystallize. Together, both solutions yielded 0.0339 g (19.1% yield) of X-ray quality yellow block crystals, the unit cell parameters of which matched those previously reported for this compound (1001174).[1]

2.6 Compound **3**, K([2.2.2]cryptand)[Er(COT)$_2$]

To a stirring suspension of $ErCl_3$ (0.0379 g, 0.1385 mmol, ca. 2 mL THF) was added dropwise a chilled solution of K_2COT (0.0505 g, 0.2770 mmol, ca. 3 mL THF). The murky, yellow reaction mixture was stirred in the freezer (-30 °C) for 24 h. Then, to a stirring solution of chilled K(2.2.2)cryptand (0.0521 g, 0.1383 mmol, ca. 2 mL THF) was added the original reaction mixture. A color change from yellow to orange was observed on addition. This reaction mixture was allowed to react in the freezer at -30°C for 24 hours. Insoluble impurities were removed *via* centrifugation and the vivid yellow-green supernatant was collected. Vivid yellow needle X-ray quality crystals were grown over the course of 4 days *via* pentane-THF vapor diffusion carried out in the freezer (-30 °C) (0.0482 g, Yield: 44.0%). CHN analysis (calculated, found) for $ErKC_{34}H_{52}N_2O_6$: C (51.62, 51.49); H (6.62, 6.79); N (3.54, 3.62).

2.7 Details of Dilution Study

Magnetic dilutions of **1**, **2** and **3** were prepared following the corresponding procedures for their undiluted parent compound, with the utilization of yttrium as the diamagnetic counterpart to erbium. Reactions were carried out using a 1:19 molar ratio of $ErCl_3$ to YCl_3 to produce a 5% molar dilution with respect to erbium. Diluted magnetic measurements were scaled to expected magnetization saturation values based on the parent concentrated compounds to yield resulting molar ratios:

The dilute reaction of **1** yielded a molar percent ratio of Er:Y of 5.6:94.1 and a mass percent ratio of 10.0:90.0.

The dilute reaction of **2** yielded a molar percent ratio of Er:Y of 4.8:95.2 and a mass percent ratio of 8.6:91.4.

The dilute reaction of **3** yielded a molar percent ratio of Er:Y of 6.4:93.6 and a mass percent ratio of 11.5:88.6.

3 CRYSTALLOGRAPHIC DETAILS

Single crystal diffraction data for **1** were collected on a Bruker Apex II-Ultra CCD with microfocus rotating anode using a Mo(Kα) radiation source. The structures were solved using direct methods via the SHELX routine and refined using full-matrix least-squares procedures with the SHELXL routine.[2-4] Olex^2 was used as a graphical front end during refinement.[5] Hydrogens were modeled using a riding model for all positions. Supplementary crystallographic data for structures collected and solved at 80 K and 200 K can be accessed from the Cambridge Crystallographic Data Center via deposition numbers: 2256231 (**1, 80 K**) and 2267662 (**1, 200 K**).

Single crystal diffraction data for **3** were collected at 200 K on a Bruker *κ* Diffractometer using a Mo(Kα) radiation source and an Apex II area detector. The structures were solved using direct methods via the SHELX routine and refined using full-matrix least-squares procedures with the SHELXL routine. Olex^2 was used as a graphical front end during refinement. Hydrogens were modeled using a riding model for all positions. Supplementary crystallographic data can be accessed from the Cambridge Crystallographic Data Center via deposition number: 2256233.

A unit cell collection of **2** at 200 K was completed on the aforementioned instruments and compared to previously published sources[1] as a means to characterize this compound. Unit cell parameters matched the expected compound; CCDC deposition number: 1001174.

In addition, the diamagnetic yttrium analogue of **1** was synthesized: **[CoCp$^*_2$][YCOT$_2$]**, with single crystal diffraction data collected at 200 K on a Bruker *κ* Diffractometer using a Mo(Kα) radiation source and an Apex II area detector. The structures were solved using direct methods via the SHELX routine and refined using full-matrix least-squares procedures with the SHELXL routine. Olex^2 was used as a graphical front end during refinement. Hydrogens were modeled using a riding model for all positions. Supplementary crystallographic data can be accessed from the Cambridge Crystallographic Data Center via deposition number: 2256232.

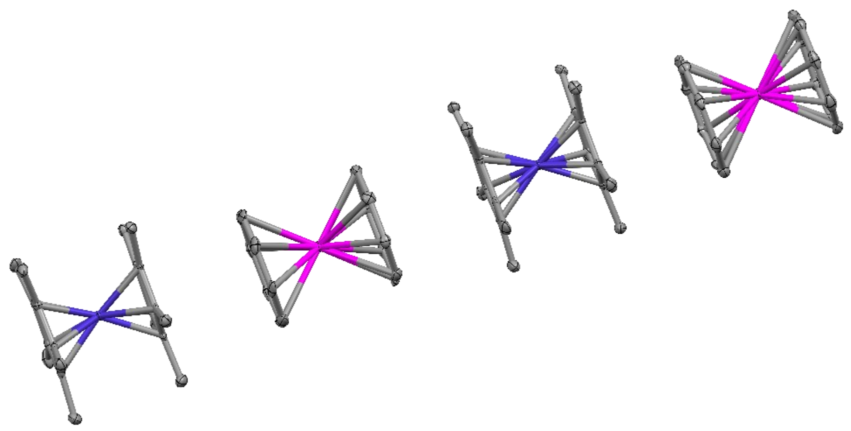

Figure S1. Crystal structure of **1, 80 K,** showing thermal ellipsoids at 50% probability. Atoms are colored by element type: gray (carbon), blue (cobalt), pink (erbium). Hydrogen atoms have been omitted for clarity.

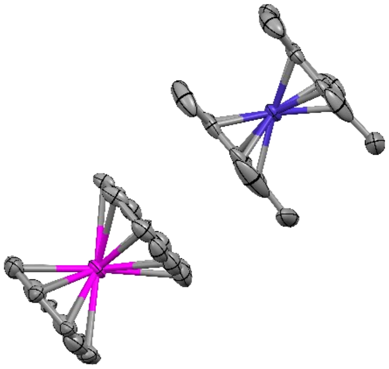

Figure S2. Crystal structure of **1, 200 K,** showing thermal ellipsoids at 50% probability. Atoms are colored by element type: gray (carbon), blue (cobalt), pink (erbium). Hydrogen atoms have been omitted for clarity.

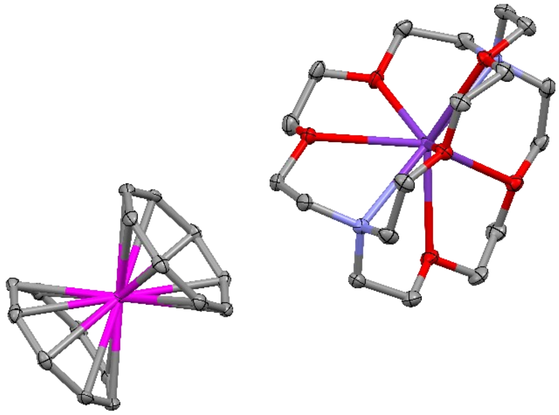

Figure S3. Crystal structure of **3**, showing thermal ellipsoids at 50% probability. Atoms are colored by element type: gray (carbon), light blue (nitrogen), pink (erbium), red (oxygen), purple (potassium). Hydrogen atoms have been omitted for clarity.

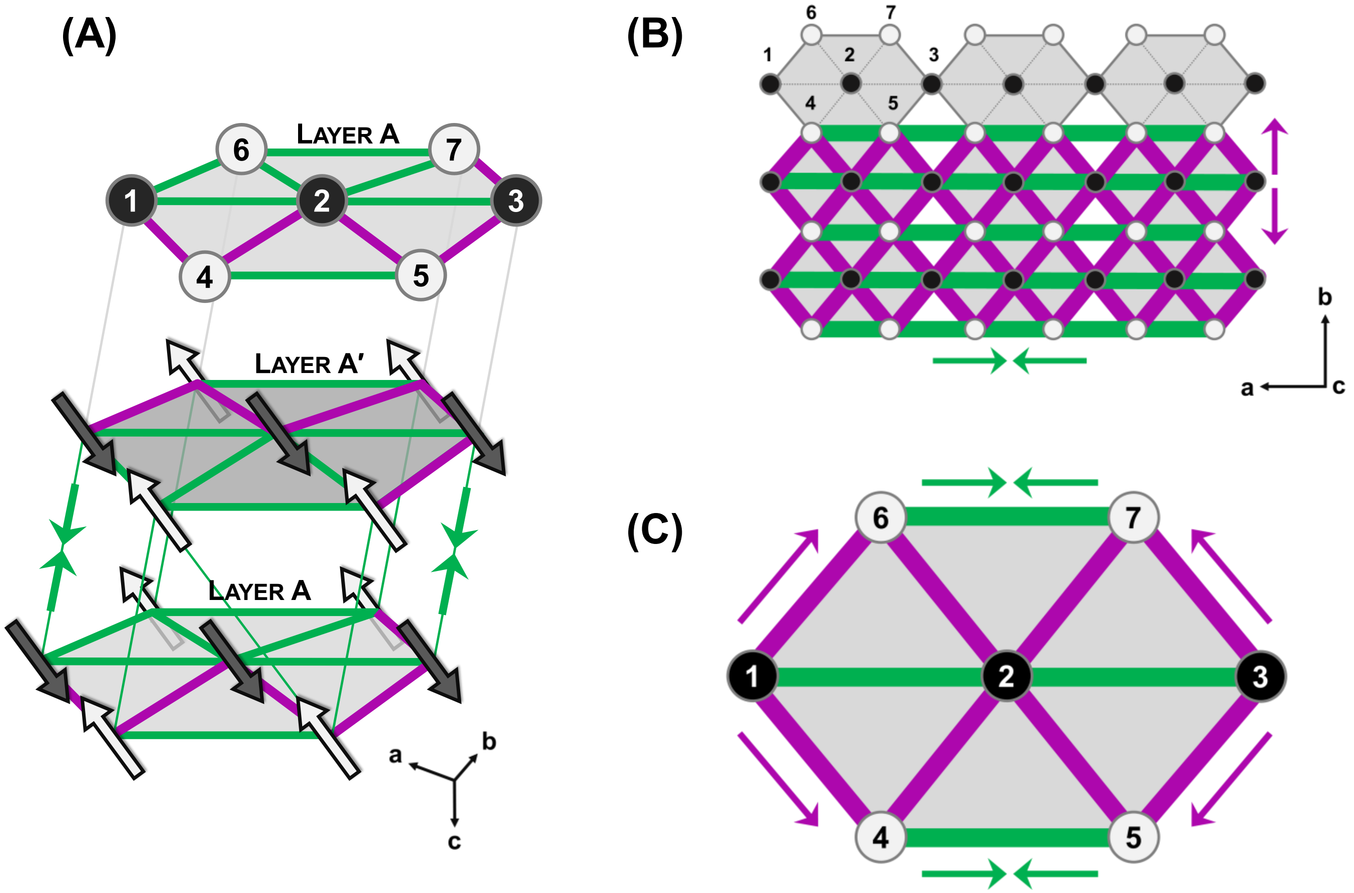

Figure S4. Crystallographically derived structural expansions (purple) and contractions (green) in **1** upon cooling from 200 K, $P2_1/m$ to 80 K, $P2_1/c$. (A) Layer-by-layer structural changes. Arrows demonstrate energy minimizing spin configuration within the local Ising axis framework. Structural changes upon cooling, in extended lattice overlay of hexagonal motifs (B) and combined across the lattice to be shown on one representative hexagonal motif (C).

4 MAGNETOMETRY DETAILS

Magnetic data were collected under DC and VSM scan modes using a Quantum Design MPMS3 SQUID Magnetometer with equipped AC susceptibility attachment. Crystal samples were finely crushed and loaded in custom quartz tubes (D&G Glassblowing Inc.), layered with eicosane wax, and subsequently flame-sealed under static vacuum. Eicosane wax was melted within the sealed sample to abate sample torquing and to facilitate thermal conductivity. Diamagnetic corrections for the samples and eicosane wax were calculated using Pascal's constants[6] and subtracted from all static moment data. Thermal magnetic susceptibilities were collected as ZFC and FC data in DC scan mode under applied fields of H = 100, 250, 500, 750, 1,000, 10,000, and 40,000 Oe. Isothermal magnetization data were collected in VSM mode between −7 to 7 T at a 60 Oe sec⁻ sweep rate for full hysteresis loops and virgin curves.

Short and long-timescale AC data were fit to a Debye (Cole-Cole relaxation) model. Details related to the collection and analysis of long-timescale magnetic data are discussed in our previous works.[7,8]

Temperature and τ data were fit to a multi-term relaxation model shown in Equation S1.

MPMS 3 data parsing, fitting, and plotting was performed with our MATLAB package, *Super*. This object-oriented code package and all applicable documentation is available at https://github.com/RinehartGroup/super-matlab under the MIT License.[9]

$$\tau^{-1} = \tau_0^{-1} exp\left(\frac{-U_{eff}}{k_B T}\right) + \tau_D^{-1} exp\left(\frac{-D_{eff}}{k_B T}\right) \quad \textbf{(Eq. S1)}$$

Equation S1. Multi-term relaxation mechanism equation accounting for Orbach and dipolar processes, where τ is the fitted relaxation time, τ_0 is the attempt time, U_{eff} is the effective barrier, k_B is the Boltzmann constant, T is the temperature, τ_D is the dipole attempt time, and D_{eff} is the dipole effective barrier.

4.1 Magnetic Data of Compounds **1** & **1-Y**

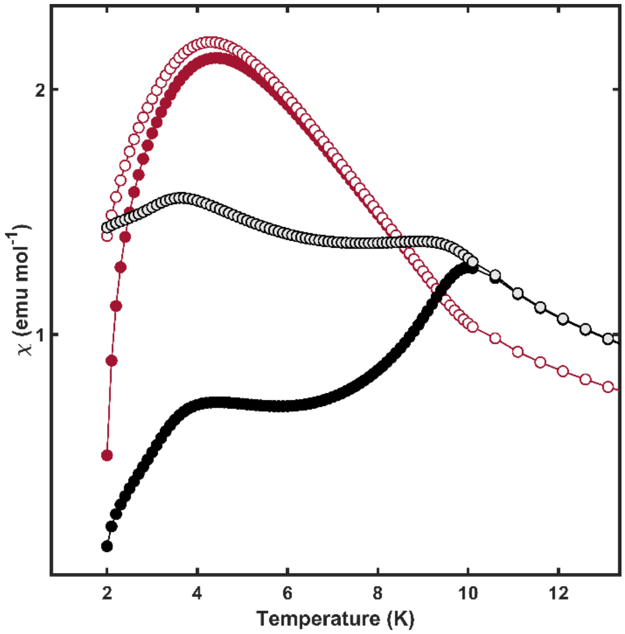

Figure S5. DC susceptibility data for **1** (colored circles) and **1-Y** (black and white circles) under H = 100 Oe applied field. ZFC data is represented by filled markers, FC by open markers.

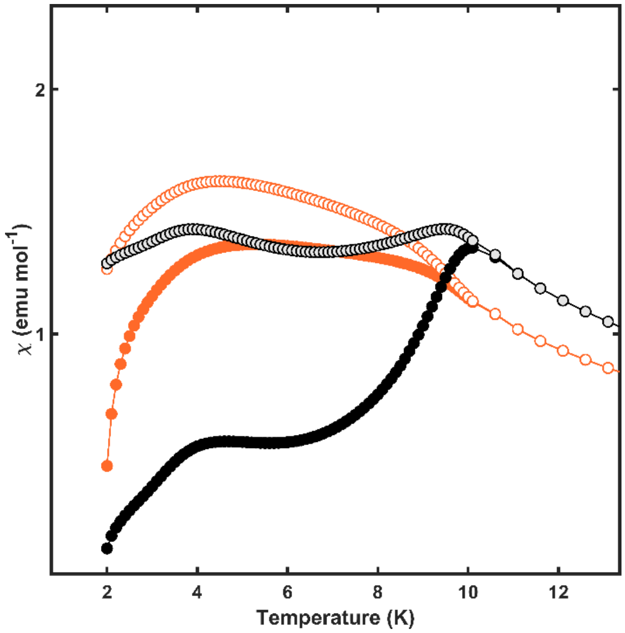

Figure S6. DC susceptibility data for **1** (colored circles) and **1-Y** (black and white circles) under H = 250 Oe applied field. ZFC data is represented by filled markers, FC by open markers.

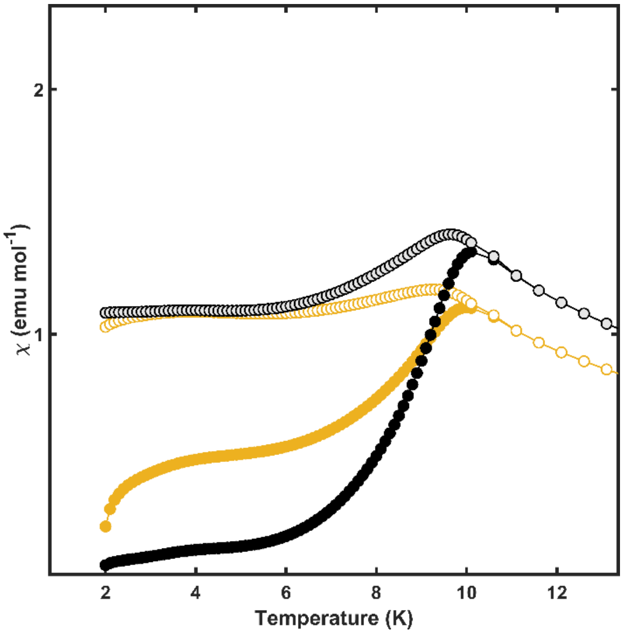

Figure S7. DC susceptibility data for **1** (colored circles) and **1-Y** (black and white circles) under H = 500 Oe applied field. ZFC data is represented by filled markers, FC by open markers.

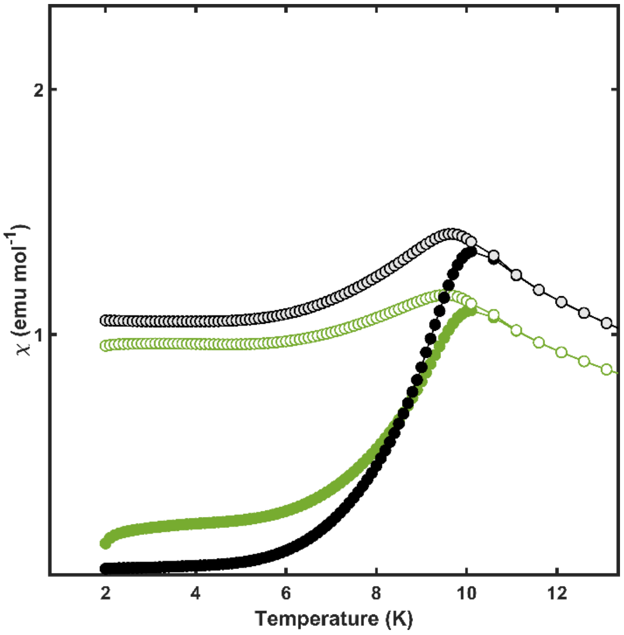

Figure S8. DC susceptibility data for **1** (colored circles) and **1-Y** (black and white circles) under H = 750 Oe applied field. ZFC data is represented by filled markers, FC by open markers.

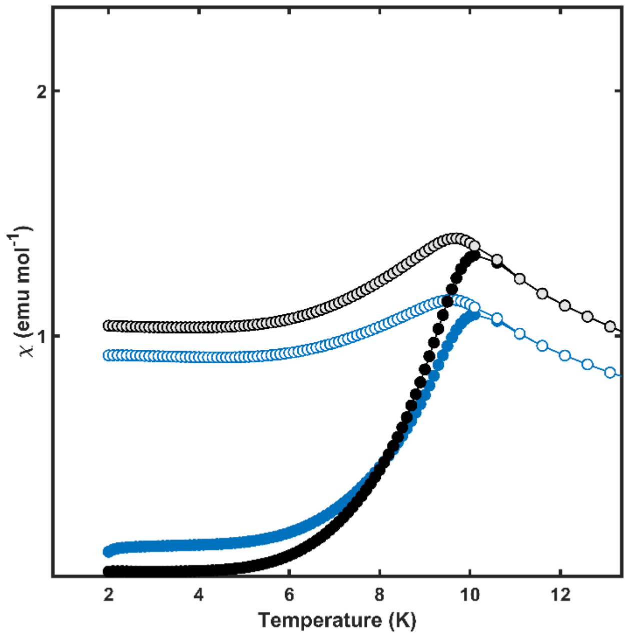

Figure S9. DC susceptibility data for **1** (colored circles) and **1-Y** (black and white circles) under H = 1,000 Oe applied field. ZFC data is represented by filled markers, FC by open markers.

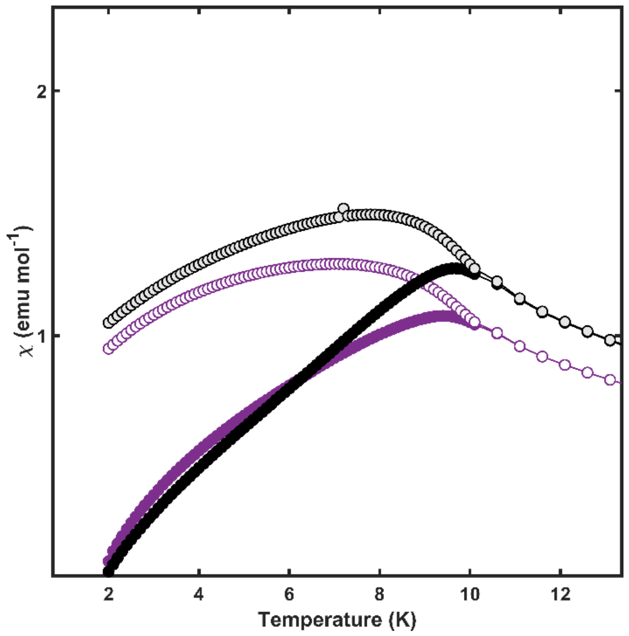

Figure S10. DC susceptibility data for **1** (colored circles) and **1-Y** (black and white circles) under H = 10,000 Oe applied field. ZFC data is represented by filled markers, FC by open markers.

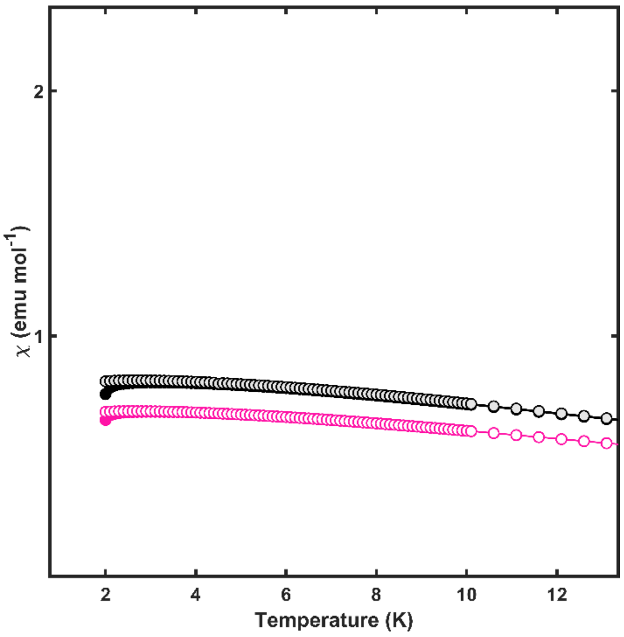

Figure S11. DC susceptibility data for **1** (colored circles) and **1-Y** (black and white circles) under H = 40,000 Oe applied field. ZFC data is represented by filled markers, FC by open markers.

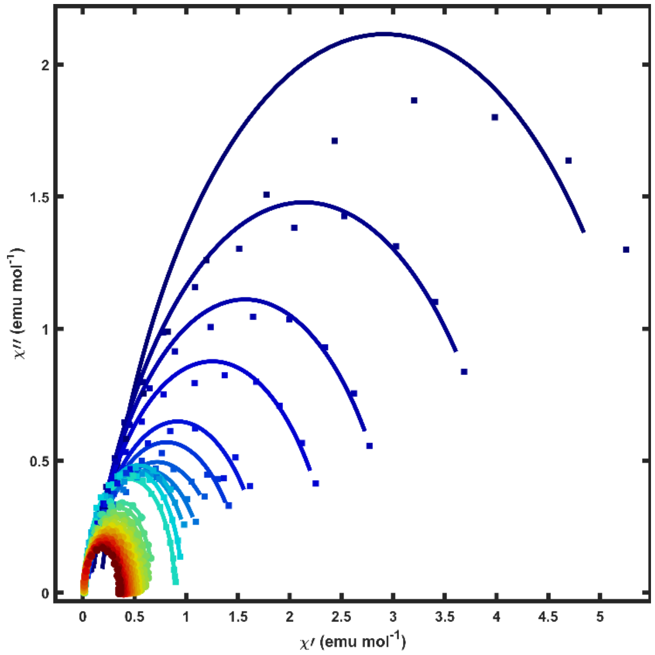

Figure S12. Cole-cole plot of **1** collected between T = 2 - 29 K (blue - red). Data points are susceptibilities measured via standard AC measurements (circles) and extracted from Fourier analysis of VSM data (squares). Lines represent fits to a generalized Debye model.

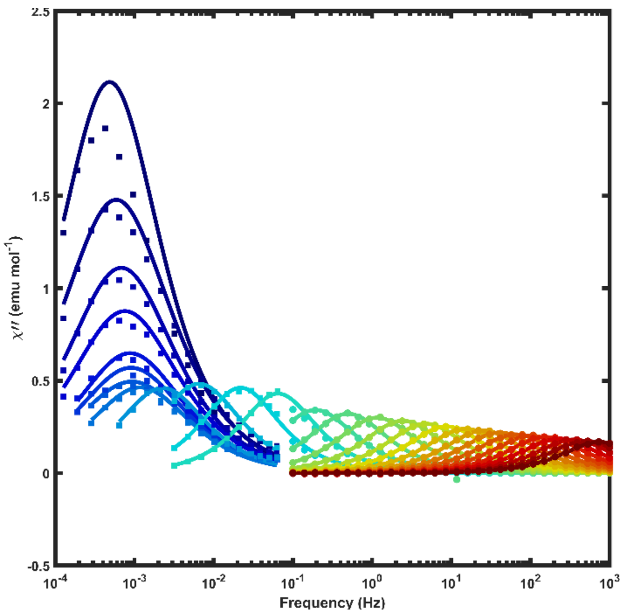

Figure S13. AC out-of-phase susceptibility (χ'') of **1** collected between T = 2 - 29 K (blue - red). Data points are susceptibilities measured via standard AC measurements (circles) and extracted from Fourier analysis of VSM data (squares). Lines represent fits to a generalized Debye model.

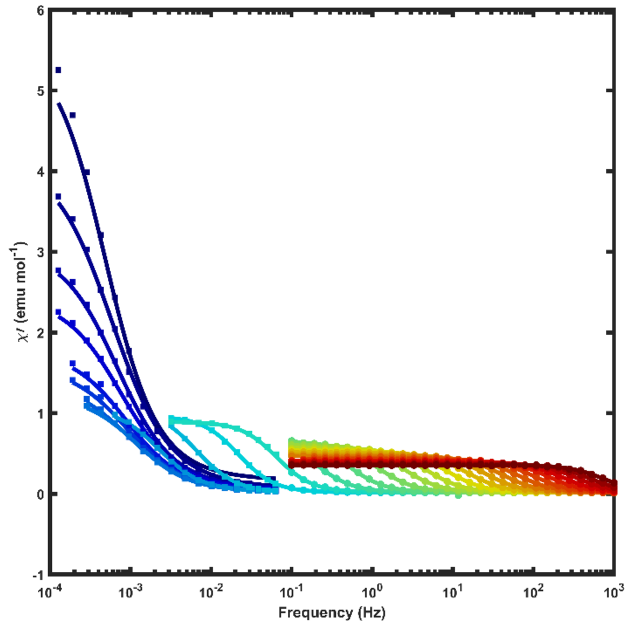

Figure S14. AC in-phase susceptibility (χ') of **1** collected between T = 2 - 29 K (blue - red). Data points are susceptibilities measured via standard AC measurements (circles) and extracted from Fourier analysis of VSM data (squares). Lines represent fits to a generalized Debye model.

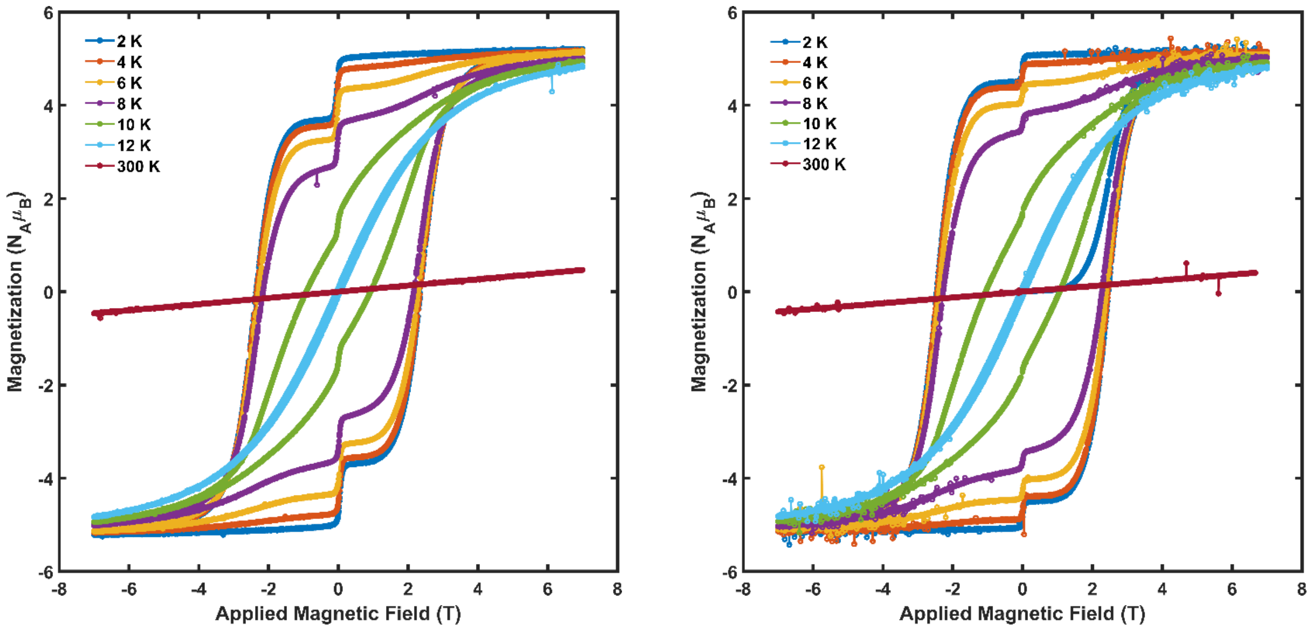

Figure S15. Isothermal magnetization of **1 (left) and 1-Y (right)** at T = 2, 4, 6, 8, 10, 12, and 300 K collected between $H = -7$ to 7 T at a constant sweep rate of 60 Oe sec^{-1}. Markers are data points from VSM mode collection, lines through the data are guides for the eye.

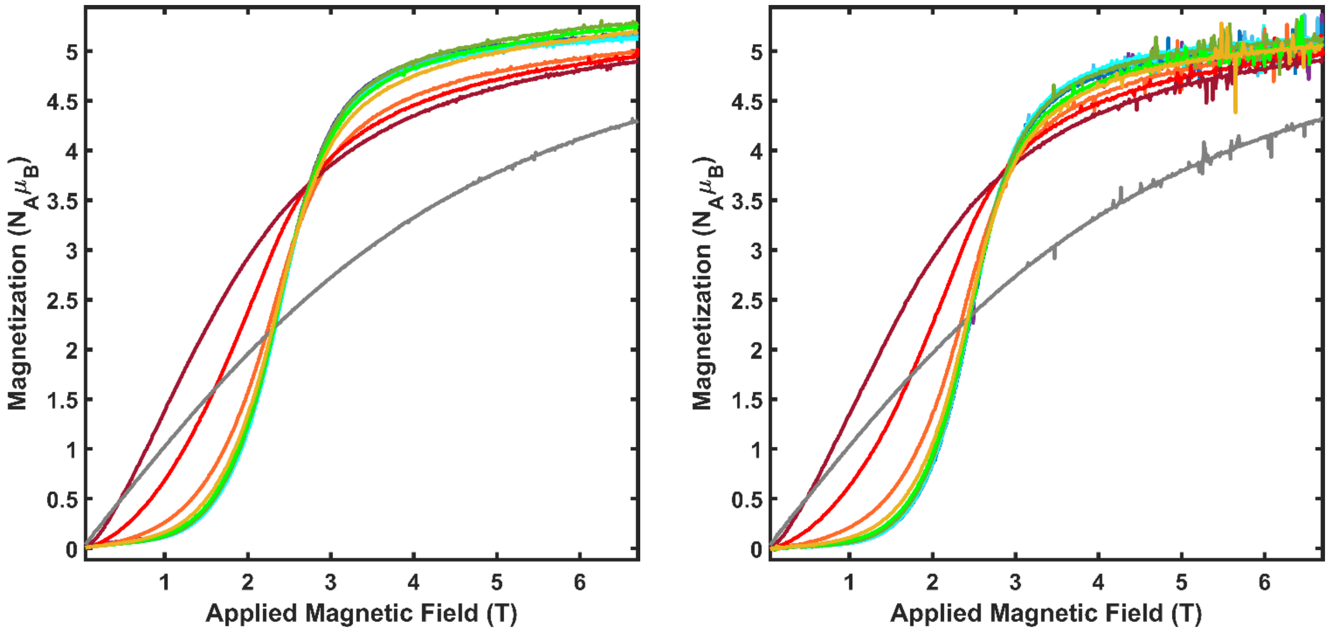

Figure S16. Virgin isothermal magnetization of **1 (left) and 1-Y (right)** at $T = 2 - 12$ K (purple to blue to red) and 20 K (gray), collected in VSM mode between $H = 0$ to 7 T at a constant sweep rate of 60 Oe sec^{-1}.

Table S1. Model fit values for AC and waveform data collected for **1** between *T* = 2 - 29 K.

T	τ_1	$\tau_{1, error, LB}$	$\tau_{1, error, UB}$	α_1	$\alpha_{1, error, LB}$	$\alpha_{1, error, UB}$	χ_T	$\chi_{T, error, LB}$	$\chi_{T, error, UB}$	χ_S	$\chi_{S, error, LB}$	$\chi_{S, error, UB}$
2	328.34	279.83	376.86	0.17	0.10	0.23	5.67	5.24	6.10	0.16	0.03	0.29
3	270.99	255.80	286.17	0.21	0.19	0.23	4.21	4.10	4.33	0.06	0.02	0.10
4	233.67	222.08	245.26	0.20	0.17	0.22	3.09	3.01	3.16	0.05	0.02	0.08
5	210.79	199.92	221.65	0.21	0.18	0.23	2.47	2.41	2.53	0.03	0.01	0.06
6	181.58	162.26	200.90	0.19	0.15	0.24	1.80	1.71	1.89	0.03	0.00	0.07
7	176.17	161.72	190.63	0.20	0.17	0.24	1.59	1.53	1.66	0.02	0.00	0.04
8	171.70	156.68	186.71	0.22	0.19	0.25	1.43	1.36	1.49	0.01	0.00	0.03
9	135.20	125.84	144.55	0.17	0.14	0.20	1.23	1.19	1.28	0.02	0.00	0.04
10	70.30	66.33	74.28	0.08	0.05	0.12	1.08	1.04	1.11	0.03	0.01	0.04
11	24.31	23.30	25.32	0.06	0.03	0.08	1.07	1.04	1.10	0.01	0.00	0.02
12	7.29	7.17	7.41	0.01	0.00	0.02	0.96	0.95	0.97	0.02	0.01	0.02
13	2.46	2.41	2.51	0.01	0.00	0.02	0.89	0.88	0.90	0.02	0.02	0.02
14	0.83	0.82	0.83	0.04	0.03	0.04	0.74	0.73	0.74	0.01	0.01	0.02
15	0.32	0.31	0.33	0.03	0.01	0.05	0.69	0.67	0.70	0.01	0.01	0.02
16	0.14	0.14	0.14	0.03	0.03	0.04	0.64	0.64	0.65	0.01	0.01	0.01
17	0.07	0.07	0.07	0.03	0.03	0.03	0.61	0.61	0.61	0.01	0.01	0.01
18	0.03	0.03	0.03	0.03	0.02	0.03	0.57	0.57	0.57	0.01	0.01	0.01
19	0.02	0.02	0.02	0.03	0.03	0.03	0.54	0.54	0.54	0.01	0.01	0.01
20	0.01	0.01	0.01	0.03	0.03	0.03	0.51	0.51	0.52	0.01	0.01	0.01
21	0.01	0.01	0.01	0.02	0.02	0.03	0.49	0.49	0.49	0.01	0.01	0.01
22	0.00	0.00	0.00	0.02	0.02	0.03	0.47	0.47	0.47	0.01	0.01	0.01
23	0.00	0.00	0.00	0.03	0.02	0.03	0.45	0.45	0.45	0.01	0.01	0.01
24	0.00	0.00	0.00	0.02	0.01	0.03	0.43	0.43	0.43	0.01	0.01	0.01
25	0.00	0.00	0.00	0.02	0.01	0.03	0.41	0.41	0.41	0.01	0.01	0.01
26	0.00	0.00	0.00	0.02	0.01	0.03	0.40	0.39	0.40	0.01	0.01	0.01
27	0.00	0.00	0.00	0.02	0.00	0.03	0.38	0.38	0.38	0.01	0.00	0.02
28	0.00	0.00	0.00	0.00	-0.01	0.02	0.37	0.37	0.37	0.02	0.01	0.03
29	0.00	0.00	0.00	0.00	-0.02	0.02	0.36	0.35	0.36	0.02	0.00	0.03

4.2 Magnetic Data of Compounds **2** & **2-Y**

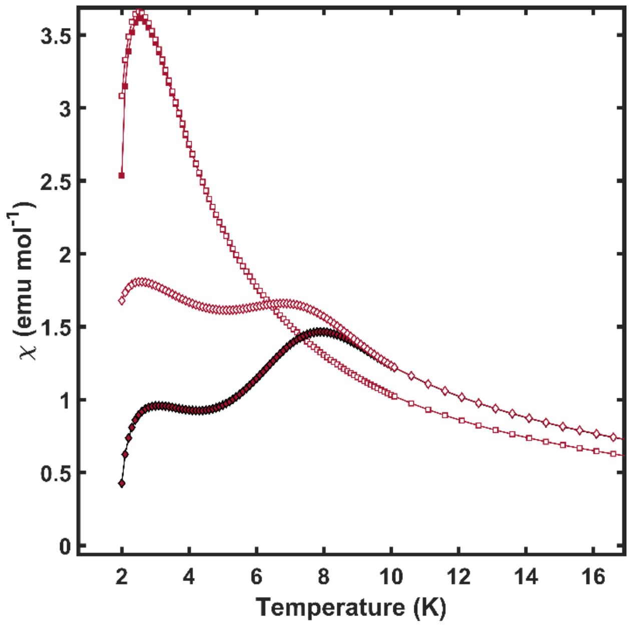

Figure S17. DC susceptibility data for **2** (squares) and **2-Y** (diamonds) under H = 100 Oe applied field. ZFC data is represented by filled markers, FC by open markers.

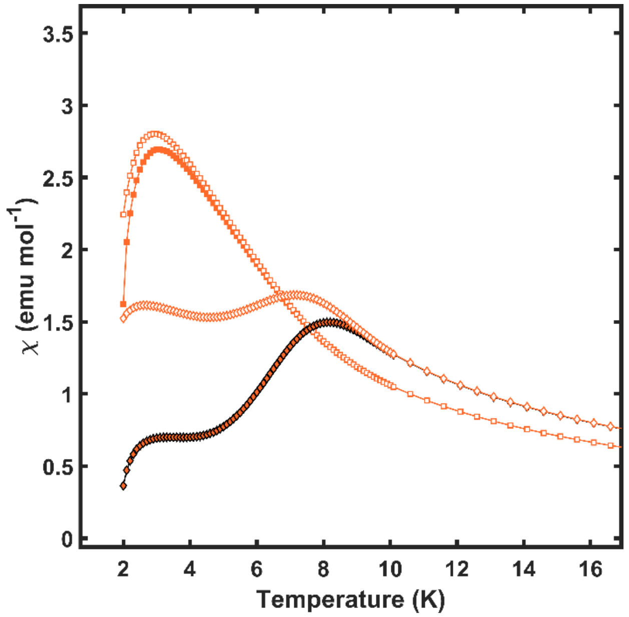

Figure S18. DC susceptibility data for **2** (squares) and **2-Y** (diamonds) under H = 250 Oe applied field. ZFC data is represented by filled markers, FC by open markers.

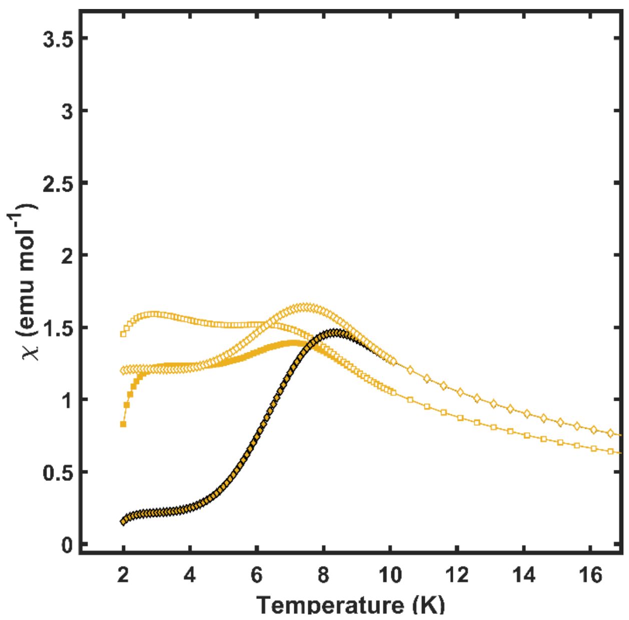

Figure S19. DC susceptibility data for **2** (squares) and **2-Y** (diamonds) under H = 500 Oe applied field. ZFC data is represented by filled markers, FC by open markers.

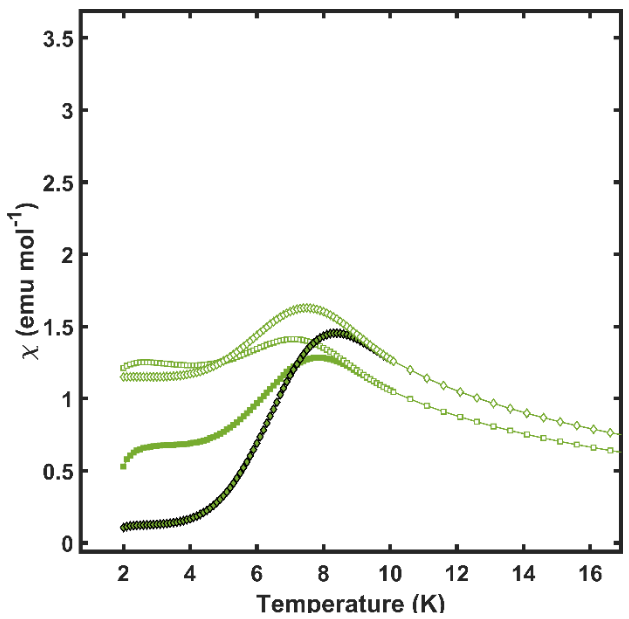

Figure S20. DC susceptibility data for **2** (squares) and **2-Y** (diamonds) under H = 750 Oe applied field. ZFC data is represented by filled markers, FC by open markers.

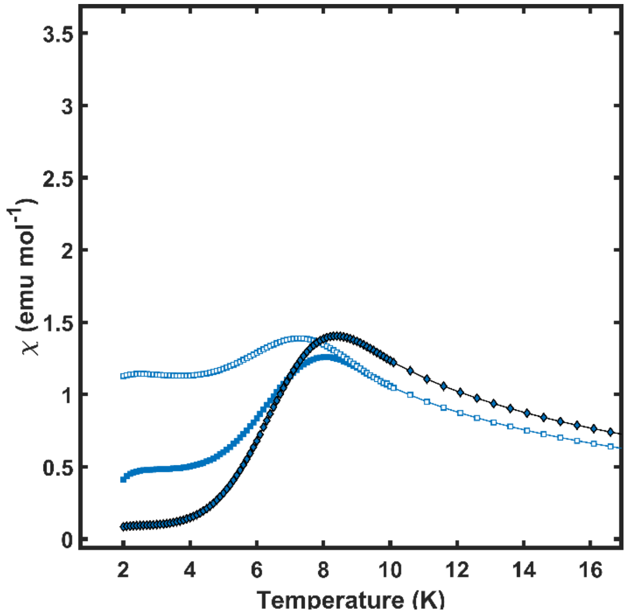

Figure S21. DC susceptibility data for **2** (squares) and **2-Y** (diamonds) under H = 1,000 Oe applied field. ZFC data is represented by filled markers, FC by open markers.

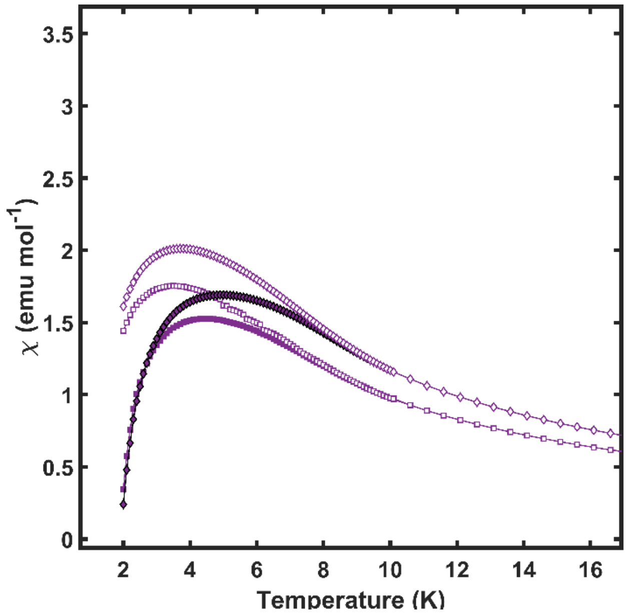

Figure S22. DC susceptibility data for **2** (squares) and **2-Y** (diamonds) under H = 10,000 Oe applied field. ZFC data is represented by filled markers, FC by open markers.

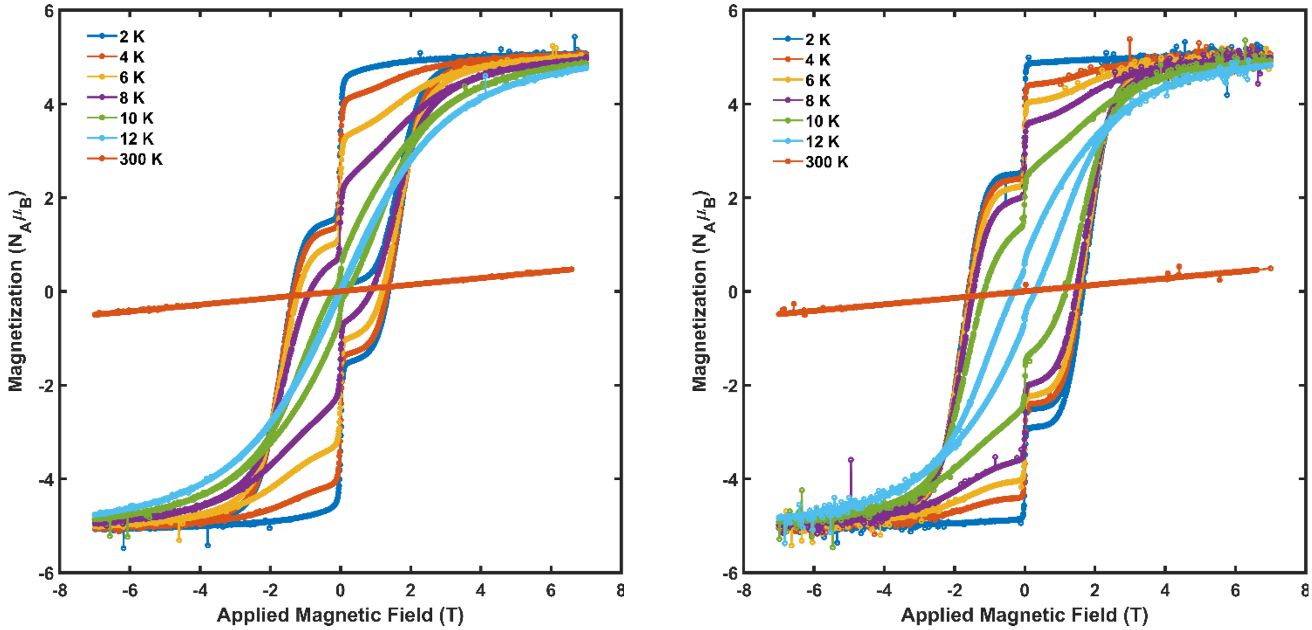

Figure S23. Isothermal magnetization of **2 (left) and 2-Y (right)** at T = 2, 4, 6, 8, 10, 12, and 300 K collected between $H = -7$ to 7 T at a constant sweep rate of 60 Oe sec^{-1}. Markers are data points from VSM mode collection, lines through the data are guides for the eye.

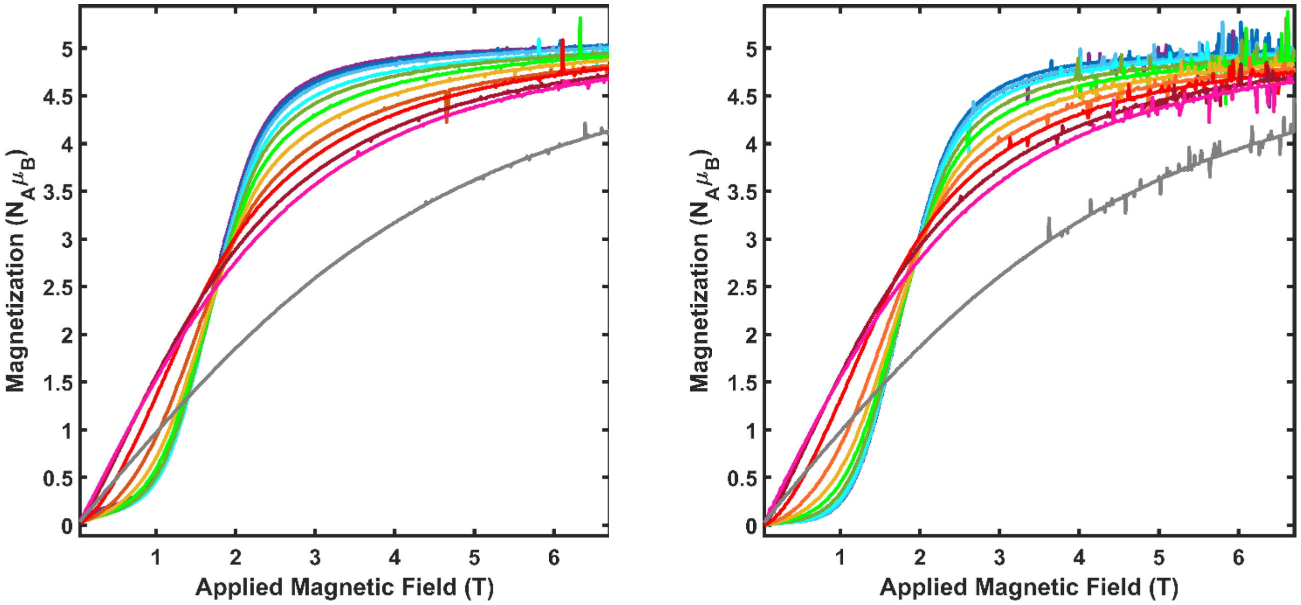

Figure S24. Virgin isothermal magnetization of **2** (left) and **2-Y** (right) at $T = 2 - 12$ K (purple to blue to red) and 20 K (gray), collected in VSM mode between $H = 0$ to 7 T at a constant sweep rate of 60 Oe sec^{-1}.

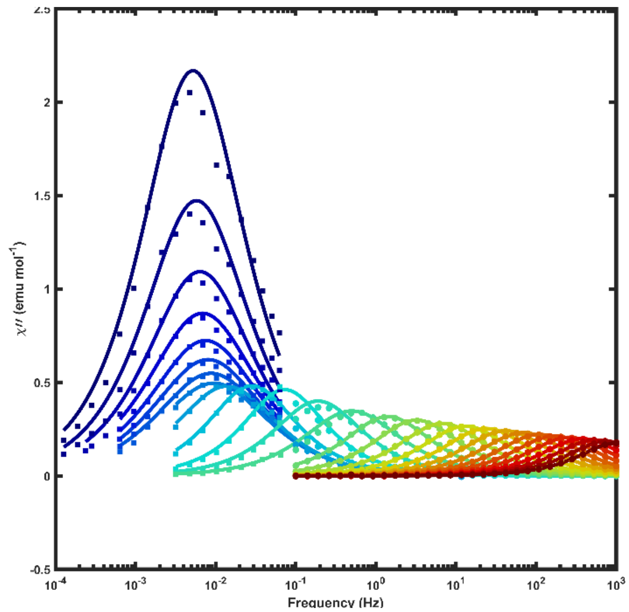

Figure S25. AC out-of-phase susceptibility (χ'') of **2** collected between T = 2 - 28 K (blue - red). Data points are susceptibilities measured via standard AC measurements (circles) and extracted from Fourier analysis of VSM data (squares). Lines represent fits to a generalized Debye model.

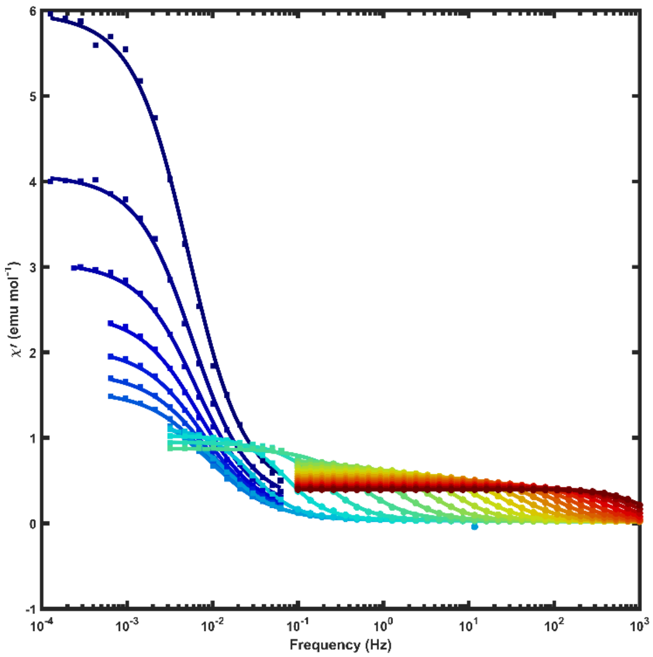

Figure S26. AC in-phase susceptibility (χ') of **2** collected between T = 2 - 28 K (blue - red). Data points are susceptibilities measured via standard AC measurements (circles) and extracted from Fourier analysis of VSM data (squares). Lines represent fits to a generalized Debye model.

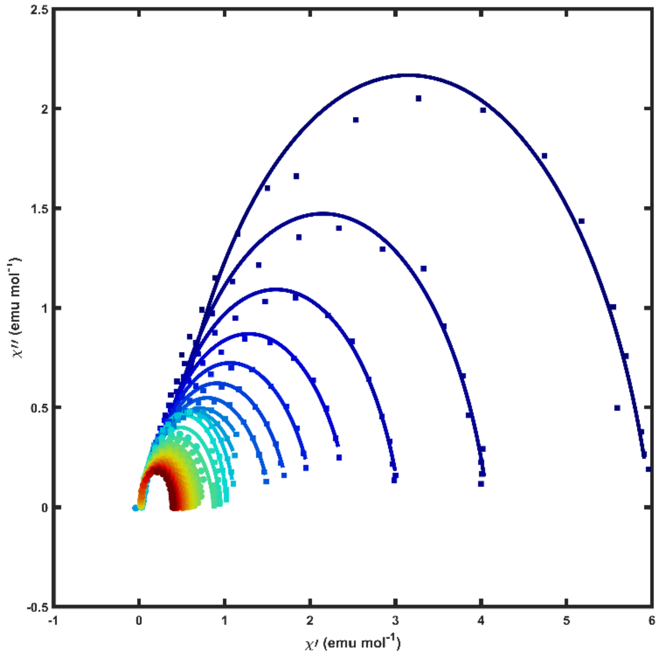

Figure S27. Cole-cole plot of **2** collected between T = 2 - 28 K (blue - red). Data points are susceptibilities measured via standard AC measurements (circles) and extracted from Fourier analysis of VSM data (squares). Lines represent fits to a generalized Debye model.

Table S2. Model fit values for AC and waveform data collected for **2** between T = 2 - 28 K.

T	τ_1	$\tau_{1, error, LB}$	$\tau_{1, error, UB}$	α_1	$\alpha_{1, error, LB}$	$\alpha_{1, error, UB}$	χ_T	$\chi_{T, error, LB}$	$\chi_{T, error, UB}$	χ_S	$\chi_{S, error, LB}$	$\chi_{S, error, UB}$
2	30.43	28.66	32.20	0.17	0.14	0.20	5.99	5.88	6.09	0.32	0.19	0.45
3	27.50	26.02	28.98	0.17	0.14	0.20	4.09	4.02	4.15	0.22	0.13	0.30
4	24.95	23.71	26.19	0.18	0.16	0.21	3.06	3.02	3.11	0.14	0.08	0.20
5	23.13	21.86	24.40	0.20	0.17	0.23	2.47	2.42	2.53	0.09	0.04	0.14
6	21.31	20.11	22.51	0.20	0.17	0.23	2.06	2.02	2.11	0.07	0.02	0.12
7	19.79	18.61	20.97	0.20	0.17	0.24	1.78	1.74	1.82	0.05	0.01	0.10
8	18.12	17.04	19.21	0.20	0.17	0.23	1.55	1.52	1.59	0.04	0.00	0.08
9	16.78	15.82	17.73	0.21	0.19	0.23	1.41	1.37	1.45	0.03	0.03	0.04
10	11.54	11.05	12.02	0.15	0.13	0.17	1.25	1.22	1.28	0.03	0.03	0.04
11	5.60	5.49	5.70	0.08	0.07	0.09	1.12	1.11	1.13	0.03	0.03	0.04
12	2.19	2.14	2.24	0.05	0.03	0.06	1.03	1.02	1.04	0.03	0.03	0.04
13	0.84	0.81	0.87	0.09	0.07	0.11	0.95	0.94	0.96	0.03	0.02	0.03
14	0.32	0.31	0.33	0.13	0.11	0.15	0.88	0.87	0.89	0.02	0.01	0.03
15	0.11	0.11	0.11	0.08	0.07	0.08	0.74	0.74	0.74	0.02	0.02	0.02
16	0.05	0.05	0.05	0.07	0.07	0.08	0.69	0.69	0.69	0.02	0.02	0.02
17	0.02	0.02	0.02	0.07	0.07	0.08	0.65	0.65	0.65	0.02	0.02	0.02
18	0.01	0.01	0.01	0.07	0.07	0.07	0.61	0.61	0.62	0.02	0.02	0.02
19	0.01	0.01	0.01	0.07	0.06	0.07	0.58	0.58	0.58	0.02	0.02	0.02
20	0.00	0.00	0.00	0.07	0.06	0.07	0.55	0.55	0.55	0.02	0.02	0.02
21	0.00	0.00	0.00	0.06	0.06	0.07	0.53	0.53	0.53	0.02	0.02	0.02
22	0.00	0.00	0.00	0.05	0.05	0.06	0.50	0.50	0.50	0.02	0.02	0.02
23	0.00	0.00	0.00	0.05	0.05	0.06	0.48	0.48	0.48	0.02	0.02	0.02
24	0.00	0.00	0.00	0.04	0.04	0.05	0.46	0.46	0.46	0.02	0.02	0.02
25	0.00	0.00	0.00	0.03	0.03	0.04	0.44	0.44	0.44	0.02	0.02	0.03
26	0.00	0.00	0.00	0.03	0.02	0.03	0.43	0.43	0.43	0.03	0.02	0.03
27	0.00	0.00	0.00	0.01	0.00	0.02	0.41	0.41	0.41	0.03	0.03	0.04
28	0.00	0.00	0.00	0.00	-0.01	0.01	0.40	0.40	0.40	0.04	0.03	0.05

4.3 Magnetic Data of Compounds **3** & **3-Y**

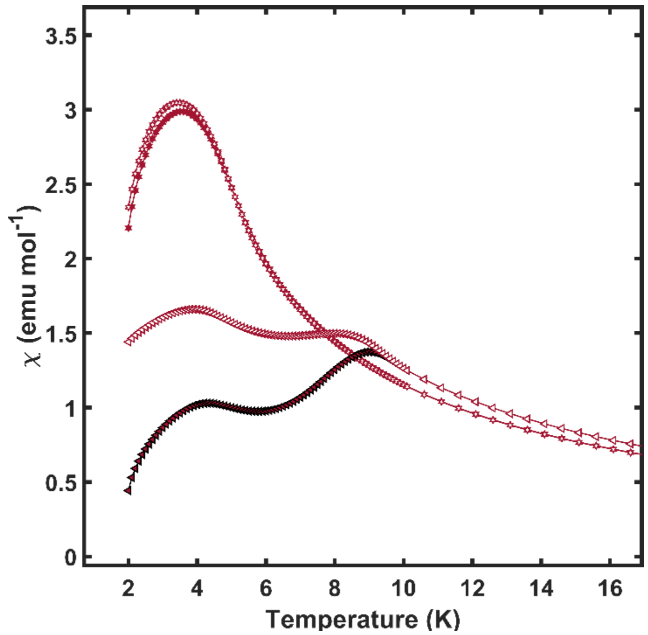

Figure S28. DC susceptibility data for **3** (stars) and **3-Y** (triangles) under H = 100 Oe applied field. ZFC data is represented by filled markers, FC by open markers.

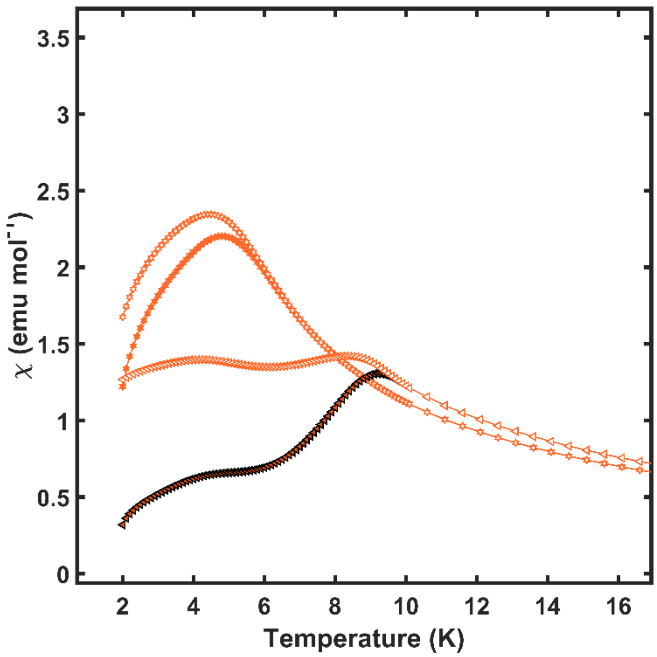

Figure S29. DC susceptibility data for **3** (stars) and **3-Y** (triangles) under H = 250 Oe applied field. ZFC data is represented by filled markers, FC by open markers.

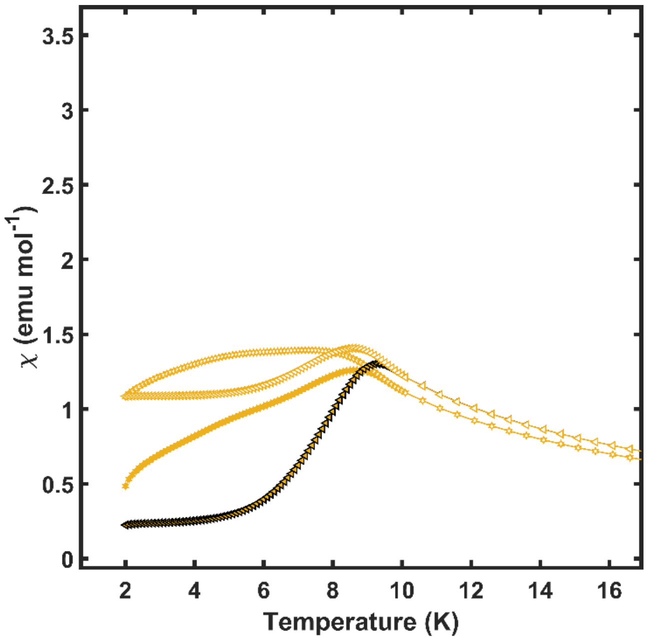

Figure S30. DC susceptibility data for **3** (stars) and **3-Y** (triangles) under H = 500 Oe applied field. ZFC data is represented by filled markers, FC by open markers.

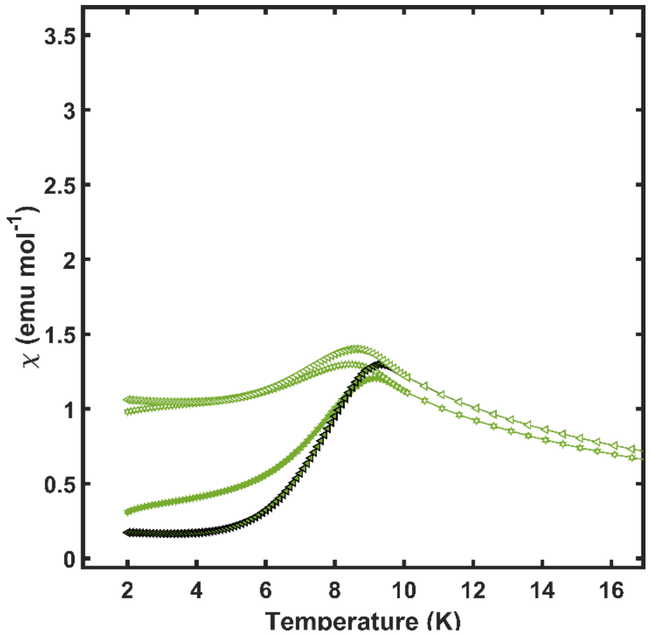

Figure S31. DC susceptibility data for **3** (stars) and **3-Y** (triangles) under H = 750 Oe applied field. ZFC data is represented by filled markers, FC by open markers.

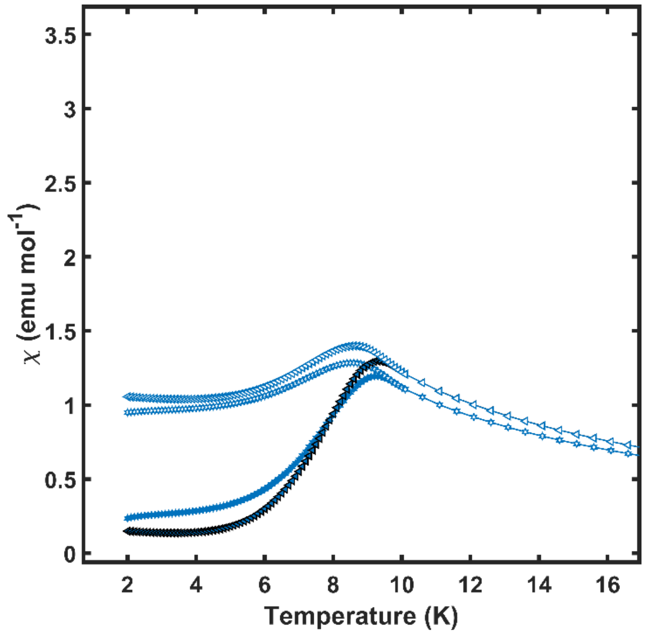

Figure S32. DC susceptibility data for **3** (stars) and **3-Y** (triangles) under H = 1,000 Oe applied field. ZFC data is represented by filled markers, FC by open markers.

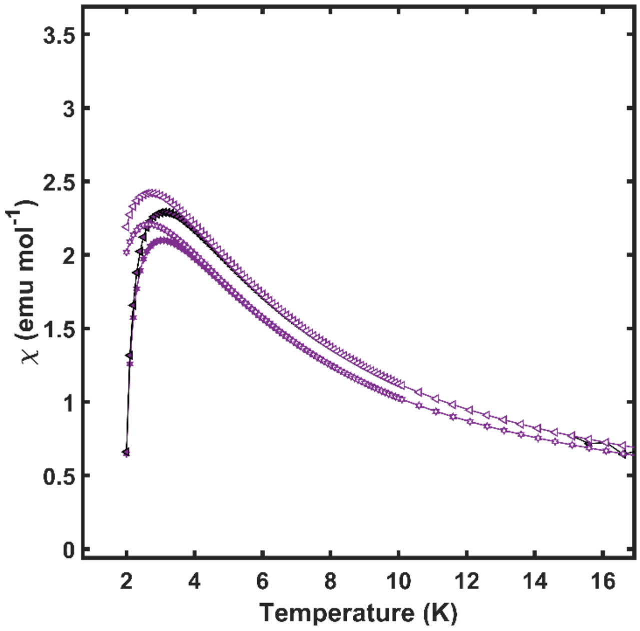

Figure S33. DC susceptibility data for **3** (stars) and **3-Y** (triangles) under H = 10,000 Oe applied field. ZFC data is represented by filled markers, FC by open markers.

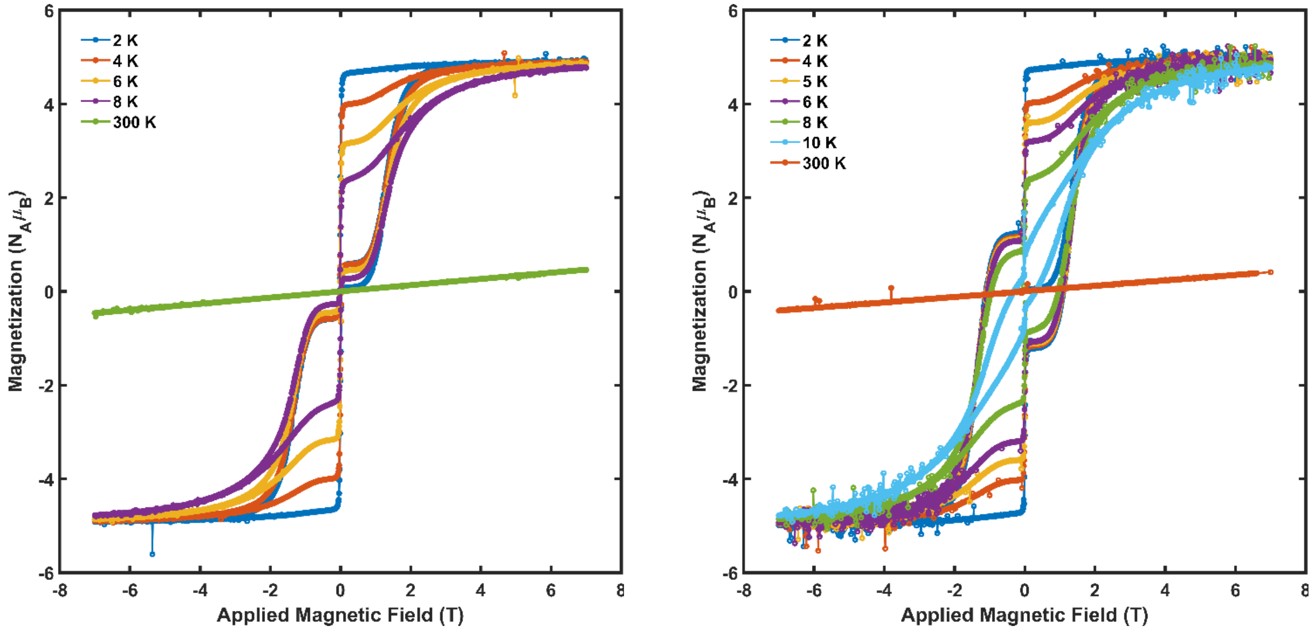

Figure S34. Isothermal magnetization of **3** (left) and **3-Y** (right) at T = 2, 4, 6, 8, 10, 12, and 300 K collected between $H = -7$ to 7 T at a constant sweep rate of 60 Oe sec^{-1}. Markers are data points from VSM mode collection, lines through the data are guides for the eye.

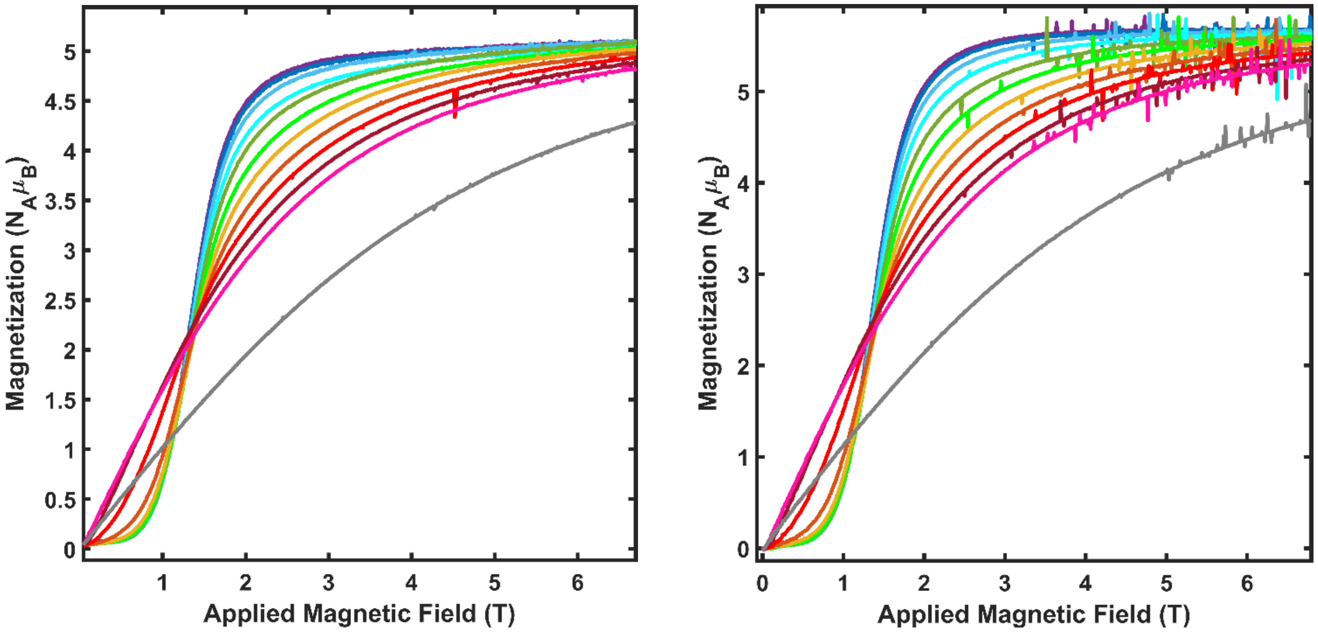

Figure S35. Virgin isothermal magnetization of **3** (left) and **3-Y** (right) at T = 2 – 12 K (purple to blue to red) and 20 K (gray), collected in VSM mode between H = 0 to 7 T at a constant sweep rate of 60 Oe sec^{-1}.

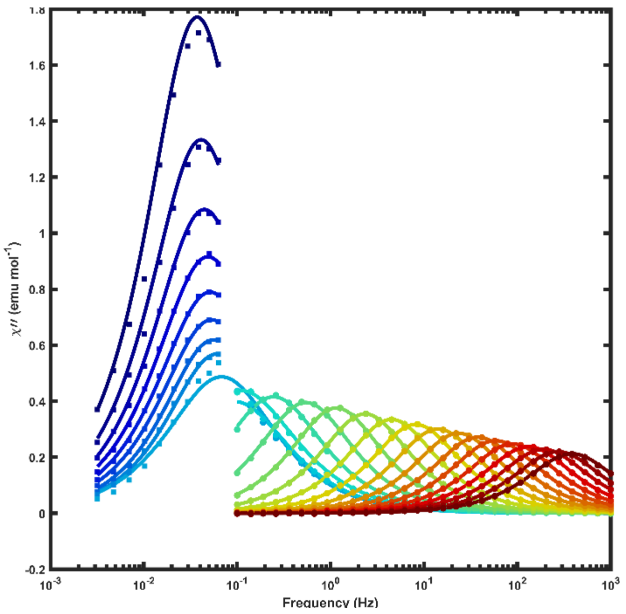

Figure S36. AC out-of-phase susceptibility (χ'') of **3** collected between T = 2 - 27 K (blue - red). Data points are susceptibilities measured via standard AC measurements (circles) and extracted from Fourier analysis of VSM data (squares). Lines represent fits to a generalized Debye model.

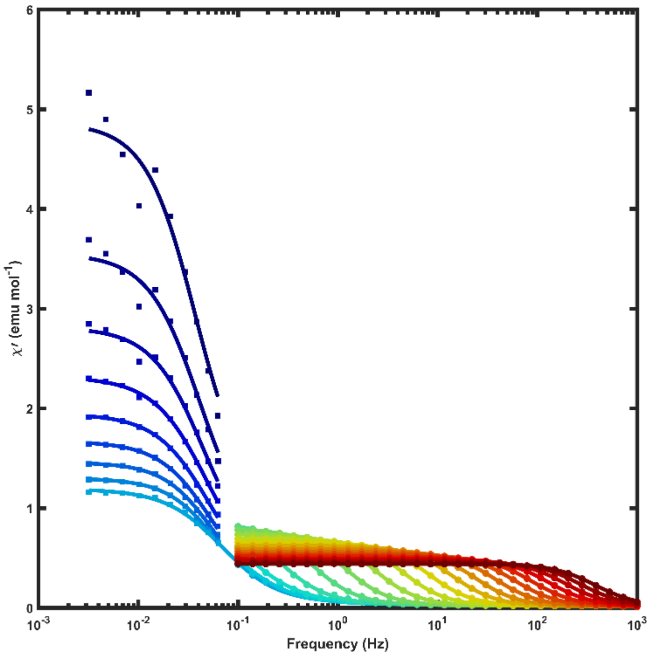

Figure S37. AC in-phase susceptibility (χ') of **3** collected between T = 2 - 27 K (blue - red). Data points are susceptibilities measured via standard AC measurements (circles) and extracted from Fourier analysis of VSM data (squares). Lines represent fits to a generalized Debye model.

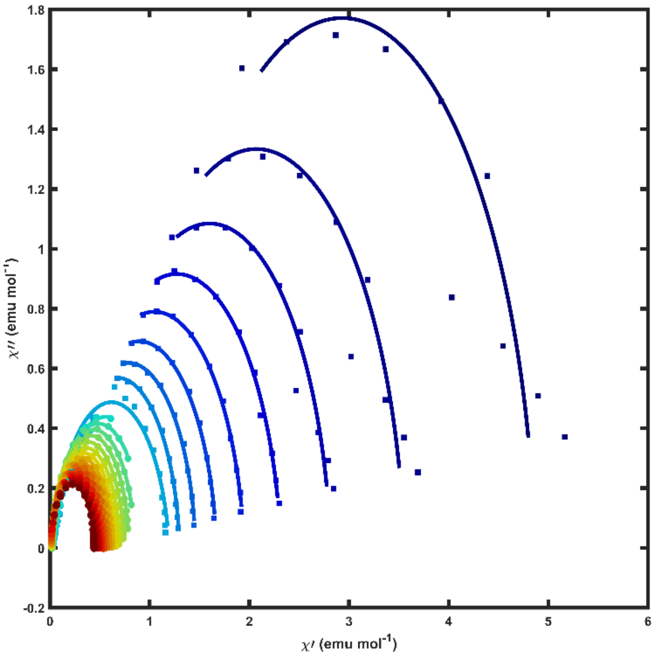

Figure S38. Cole-cole plot of **3** collected between *T* = 2 - 27 K (blue - red). Data points are susceptibilities measured via standard AC measurements (circles) and extracted from Fourier analysis of VSM data (squares). Lines represent fits to a generalized Debye model.

Table S3. Model fit values for AC and waveform data collected for **3** between T = 2 - 27 K.

T	τ_1	$\tau_{1, error, LB}$	$\tau_{1, error, UB}$	α_1	$\alpha_{1, error, LB}$	$\alpha_{1, error, UB}$	χ_T	$\chi_{T, error, LB}$	$\chi_{T, error, UB}$	χ_S	$\chi_{S, error, LB}$	$\chi_{S, error, UB}$
2	4.26	3.27	5.25	0.06	-0.08	0.19	4.87	4.62	5.12	1.00	0.31	1.68
3	3.87	3.13	4.60	0.07	-0.03	0.17	3.56	3.42	3.70	0.58	0.16	1.00
4	3.57	3.12	4.02	0.07	0.01	0.14	2.82	2.75	2.88	0.38	0.16	0.61
5	3.26	3.09	3.44	0.09	0.06	0.11	2.32	2.30	2.34	0.22	0.14	0.30
6	3.09	2.92	3.26	0.09	0.06	0.11	1.95	1.93	1.97	0.15	0.07	0.22
7	2.99	2.79	3.18	0.08	0.05	0.10	1.67	1.66	1.69	0.12	0.04	0.19
8	2.87	2.70	3.05	0.05	0.03	0.08	1.46	1.45	1.47	0.11	0.05	0.17
9	2.62	2.46	2.77	0.06	0.04	0.08	1.30	1.29	1.31	0.05	0.00	0.11
10	2.34	2.26	2.41	0.11	0.09	0.13	1.20	1.18	1.21	0.04	0.03	0.04
11	1.67	1.49	1.86	0.11	0.08	0.14	0.98	0.91	1.05	0.03	0.03	0.03
12	1.45	1.38	1.51	0.13	0.11	0.14	1.10	1.07	1.13	0.03	0.02	0.03
13	0.65	0.64	0.66	0.06	0.05	0.07	0.94	0.93	0.95	0.03	0.02	0.03
14	0.29	0.29	0.29	0.03	0.02	0.03	0.85	0.85	0.85	0.02	0.02	0.02
15	0.14	0.14	0.14	0.01	0.01	0.02	0.79	0.79	0.79	0.02	0.02	0.02
16	0.07	0.07	0.07	0.01	0.01	0.01	0.74	0.74	0.74	0.02	0.02	0.02
17	0.04	0.04	0.04	0.01	0.00	0.01	0.70	0.69	0.70	0.02	0.02	0.02
18	0.02	0.02	0.02	0.00	0.00	0.01	0.66	0.66	0.66	0.02	0.02	0.02
19	0.01	0.01	0.01	0.00	0.00	0.01	0.62	0.62	0.62	0.02	0.02	0.02
20	0.01	0.01	0.01	0.00	0.00	0.01	0.59	0.59	0.59	0.02	0.02	0.02
21	0.00	0.00	0.00	0.01	0.00	0.01	0.56	0.56	0.56	0.02	0.02	0.02
22	0.00	0.00	0.00	0.01	0.00	0.01	0.54	0.54	0.54	0.01	0.01	0.02
23	0.00	0.00	0.00	0.01	0.00	0.01	0.52	0.51	0.52	0.01	0.01	0.02
24	0.00	0.00	0.00	0.01	0.00	0.01	0.49	0.49	0.49	0.01	0.01	0.02
25	0.00	0.00	0.00	0.01	0.00	0.01	0.47	0.47	0.48	0.01	0.01	0.02
26	0.00	0.00	0.00	0.00	0.00	0.01	0.46	0.46	0.46	0.02	0.01	0.02
27	0.00	0.00	0.00	0.00	-0.01	0.01	0.44	0.44	0.44	0.01	0.01	0.02

5 FT-IR SPECTRA

Baseline-corrected solid-state FT-IR spectra were collected on finely ground crystalline samples with a Bruker ALPHA II diamond-anvil ATR spectrometer (32 scan average, 4 cm^{-1} resolution) under an atmosphere of dinitrogen.

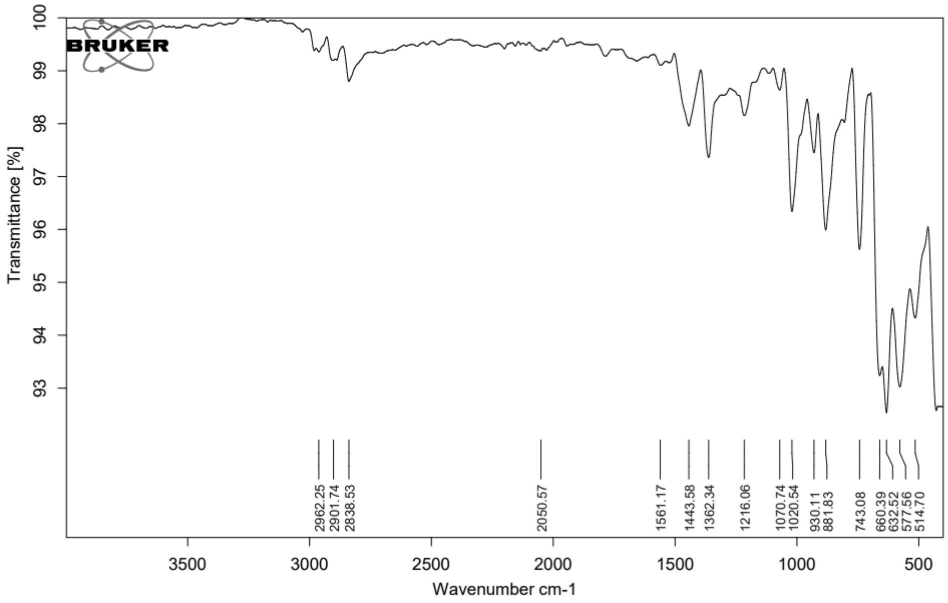

Figure S39. Solid-state FT-IR spectrum of **1**.

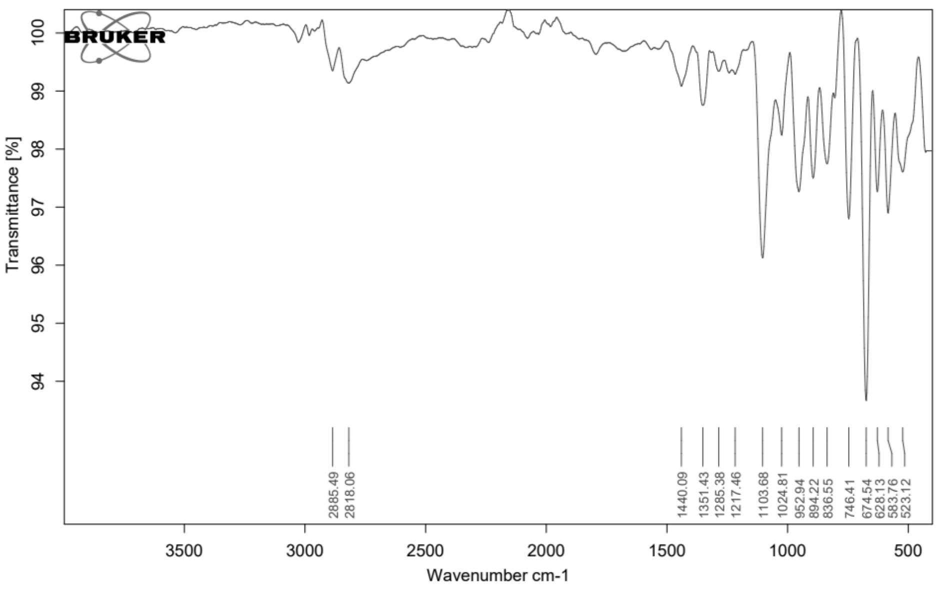

Figure S40. Solid-state FT-IR spectrum of **2**.

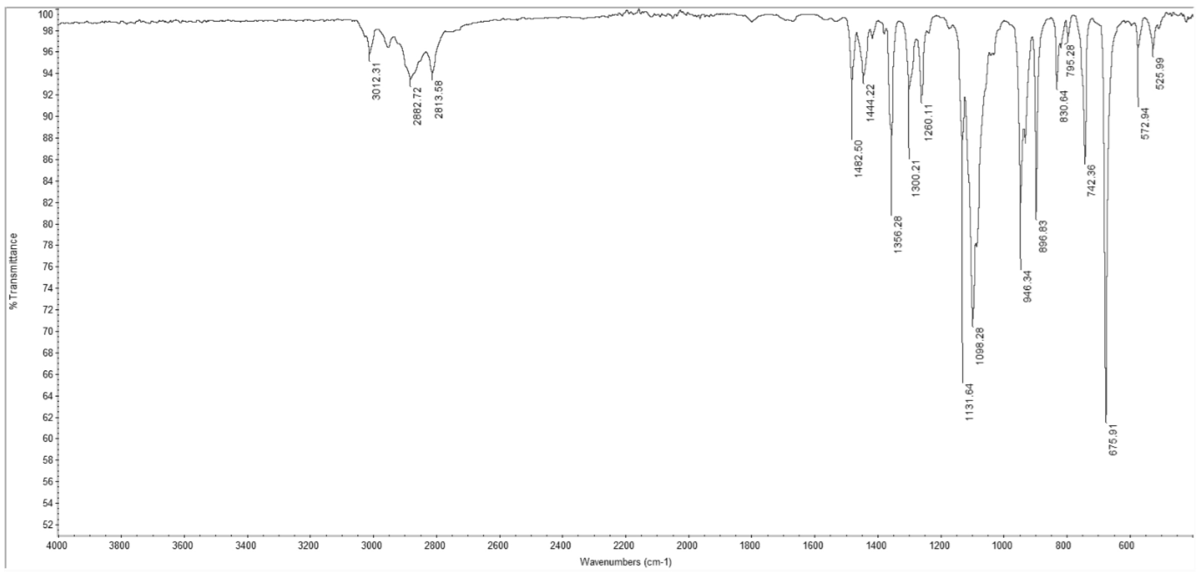

Figure S41. Solid-state FT-IR spectrum of **3**.

6 COMPUTATIONAL DETAILS

All calculations were carried out at the CASSCF level using the OpenMolcas computational package.[10,11] Input atom coordinates were taken from crystallographic data and used without further geometry optimization. Basis functions of the ANO-RCC type were generated with the SEWARD module. The quality of a specific atomic basis function was determined by the atom's connectivity to the Er^{3+} ion (**Er**: ANO-RCC-VTZP; **atoms bound to Er**: ANO-RCC-VDZP; **all other atoms**: ANO-RCC-VDZ). Two-electron integrals were Cholesky decomposed (10^{-6} cutoff). A 7-orbital, 11-electron active space (CAS(11,7)) was selected for the CASSCF calculation, which was carried out using the RASSCF module. In this space, all 35 configuration-interaction (CI) roots of spin multiplicity 4 and all 112 CI roots of spin multiplicity 2 were included. The RASSI module was used to calculate spin-orbit matrix elements between CAS output wavefunctions. The SINGLE_ANISO module of OpenMolcas was used to calculate relevant magnetic properties based on these multiconfigurational SCF results.

Table S4. CASSCF parameters of **1**, **2**, and **3** calculated with and without charge-balancing cation. Strikethrough implies calculation completed without charge-balancing cation.

Compound	KD_0, % pure, E (cm^{-1})	KD_1, % pure, E (cm^{-1})	G_x	G_y	G_z
$[CoCp^*_2][ErCOT_2]$	±15/2, 100, 0	±13/2, 99.8, 157	0.00000859424658	0.00001044867142	17.99084348335763
~~$[CoCp^*_2]$~~$[ErCOT_2]$	±15/2, 100, 0	±13/2, 100, 158	0.00000180716311	0.00000188556934	17.99196343874752
[K-18C6]$[ErCOT_2]$	±15/2, 100, 0	±13/2, 99.8, 163	0.00000641824794	0.00000844882880	17.99661106484002
~~[K-18C6]~~$[ErCOT_2]$	±15/2, 99.9, 0	±13/2, 99.9, 164	0.00000678649985	0.00000890859666	17.99420531284822
[K-k222]$[ErCOT_2]$	±15/2, 100, 0	±13/2, 99.9, 157	0.00000306657380	0.00000517739152	17.99399046042119
~~[K-k222]~~$[ErCOT_2]$	±15/2, 100, 0	±13/2, 100, 158	0.00000596522543	0.00000668636035	17.99423191521609

7 CURVE FITTING AND ANALYSIS

Overview:

A quantitative peak fitting analysis of isothermal magnetization curves involved fitting the data to a modified Cauchy Distribution. This fitting process parameterized the magnetization curves to enable a comparative analysis.[12] The modified Cauchy Distribution fits allow for deconvolution of multiple magnetic phases and/or transitions, providing insights into each magnetic transition through statistical values. Parameters such as peak width, intensity, location, and percentage based on the area under the curve are reported. Notably, this analysis condenses the magnetization curve into its gamma parameter, representing the half-width at half-maximum of the peak. Conclusions about the nature of the process occurring at that magnetic field can be drawn based on these statistical values.

Methods:

For each sample, magnetization curves were normalized to the maximum magnetization at 2 K. For all samples and temperatures, three inflection points (peaks) were detected. The data were fit to a combination of three unique Cauchy cumulative distribution functions (CDF), Equation S2. To better illustrate the temperature dependency and subtle variations in peaks, the fit parameters were applied to the probability density function (PDF) formulation of the Cauchy distribution and plotted utilizing open-access software *multi_Cauchy*.[13] The *multi_Cauchy* code package and all applicable documentation is available at https://doi.org/10.5281/zenodo.8299498, under the MIT license.

$$M(H;\ H_P, \gamma\ ) = \frac{2M_S}{\pi} arctan\left(\frac{H - H_P}{\gamma}\right) \quad \textbf{(Eq. S2)}$$

Equation S2. Modified Cauchy distribution function (CDF), where H is the applied field, H_P is the field at which a particular demagnetization (peak) maximum occurs, γ is the broadness parameter, and M_S is the saturation magnetization.

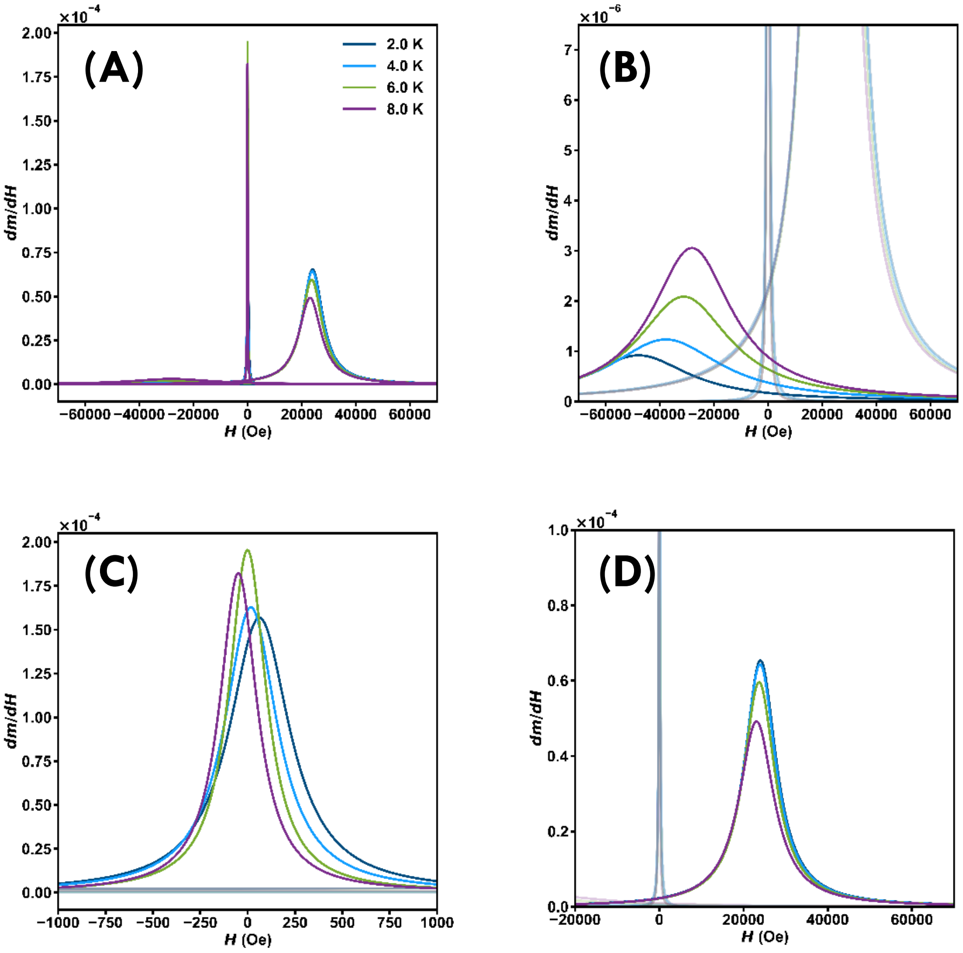

Figure S42. Cauchy fits of isothermal magnetization loops collected at T = 2, 4, 6, and 8 K of **1**. (a) Fits of all three processes. (b) Zoom of process 1. (c) Zoom of process 2. (d) Zoom of process 3.

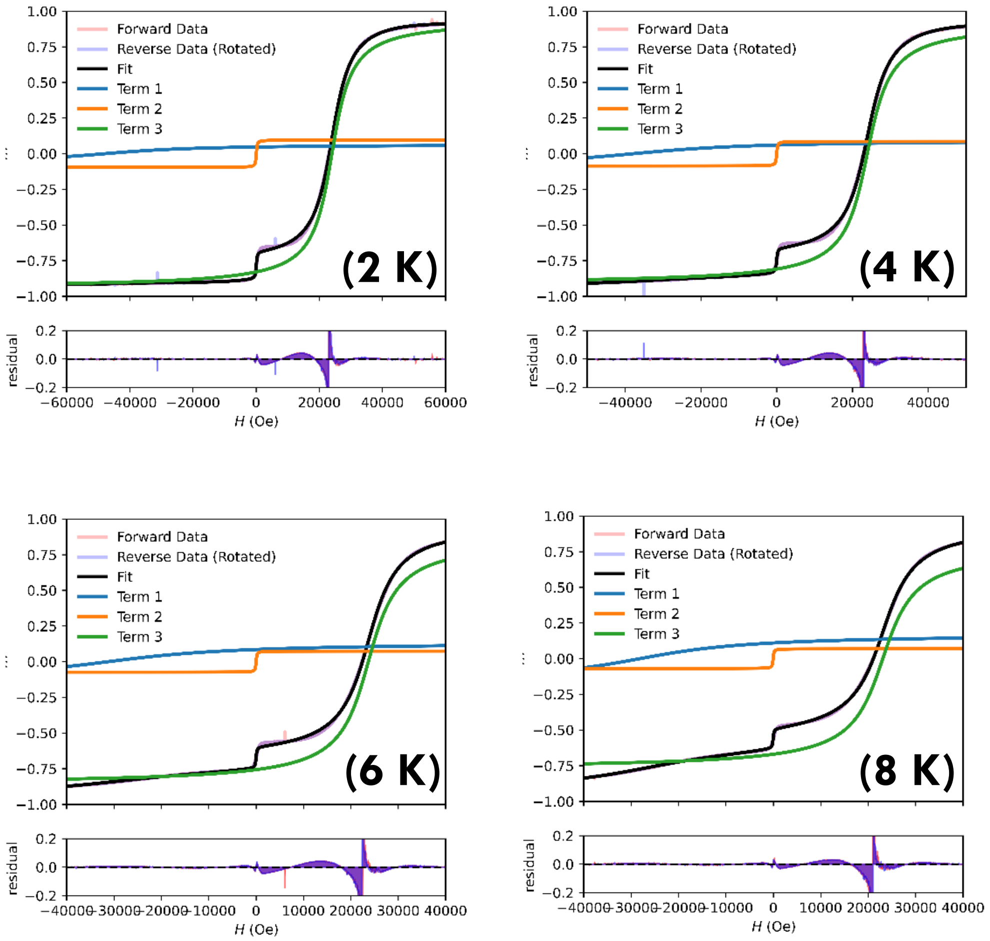

Figure S43. Cauchy fits of isothermal magnetization loops collected at T = 2, 4, 6, and 8 K of **1**, showing contributions of each term (process). Residuals are demonstrated below each graph.

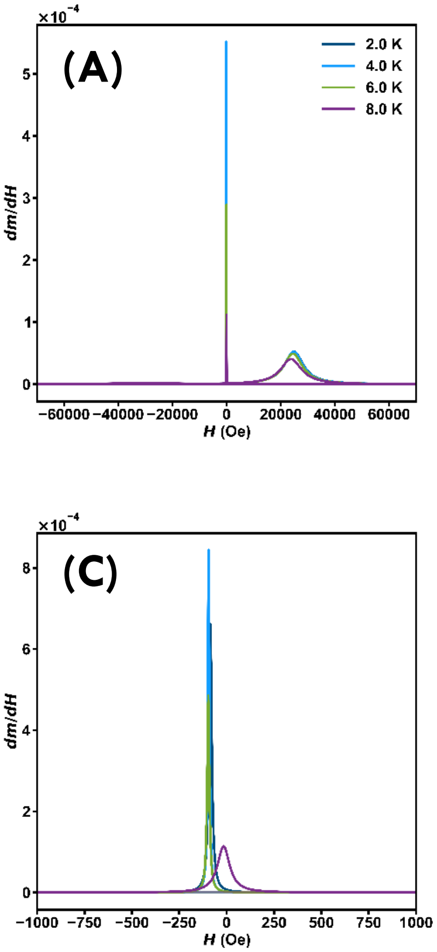

Figure S44. Cauchy fits of isothermal magnetization loops collected at T = 2, 4, 6, and 8 K of **1-Y**. (a) Fits of all three processes. (b) Zoom of process 1. (c) Zoom of process 2. (d) Zoom of process 3.

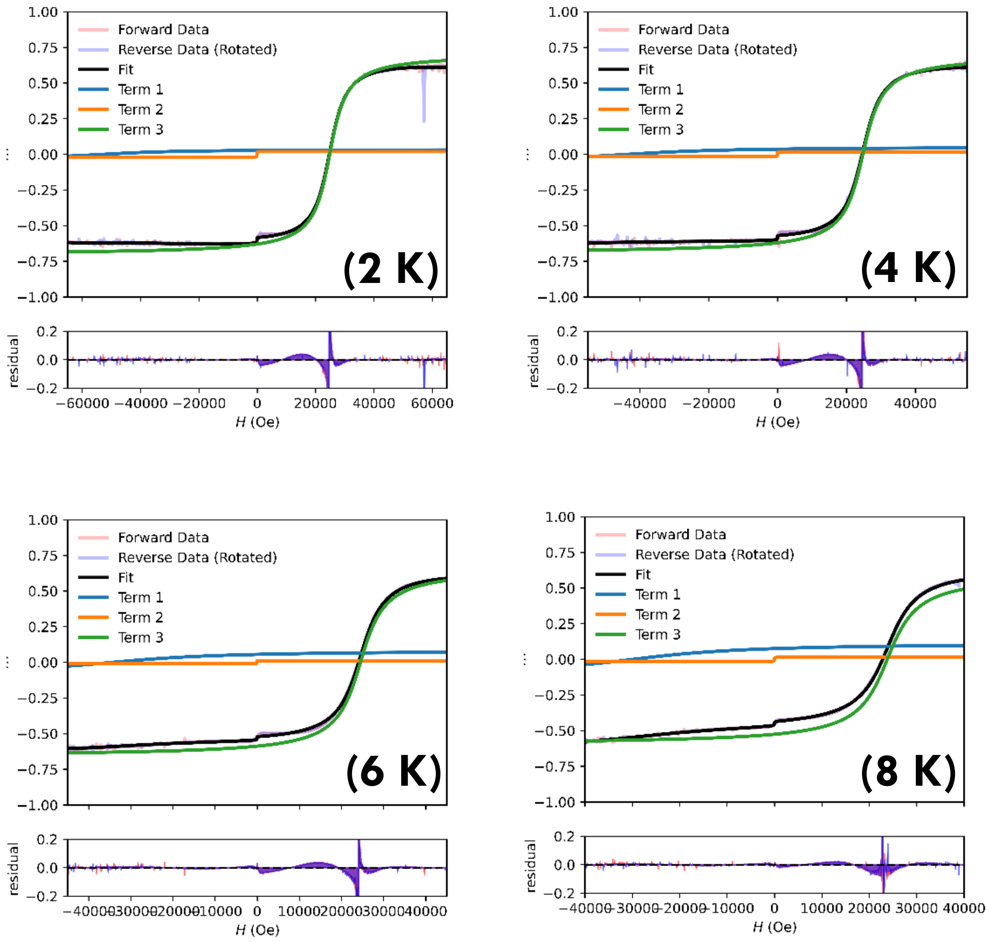

Figure S45. Cauchy fits of isothermal magnetization loops collected at T = 2, 4, 6, and 8 K of **1-Y**, showing contributions of each term (process). Residuals are demonstrated below each graph.

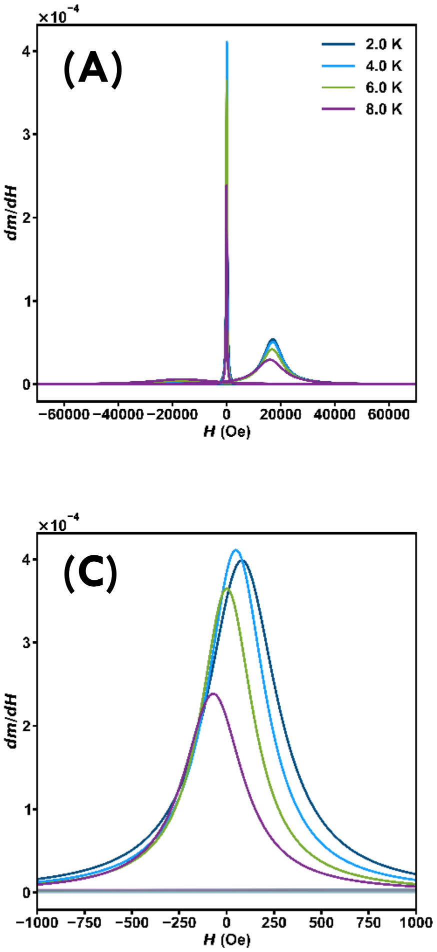

Figure S46. Cauchy fits of isothermal magnetization loops collected at $T = 2$, 4, 6, and 8 K of **2**. (a) Fits of all three processes. (b) Zoom of process 1. (c) Zoom of process 2. (d) Zoom of process 3.

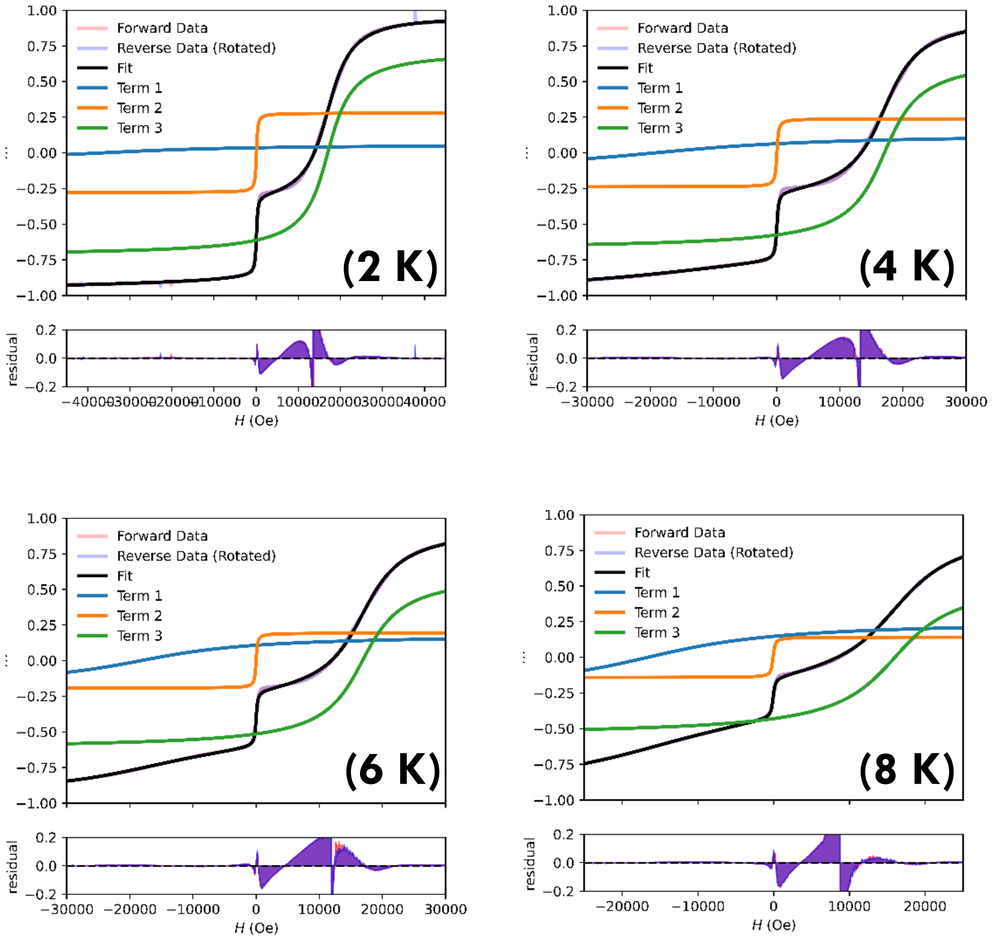

Figure S47. Cauchy fits of isothermal magnetization loops collected at T = 2, 4, 6, and 8 K of **2**, showing contributions of each term (process). Residuals are demonstrated below each graph.

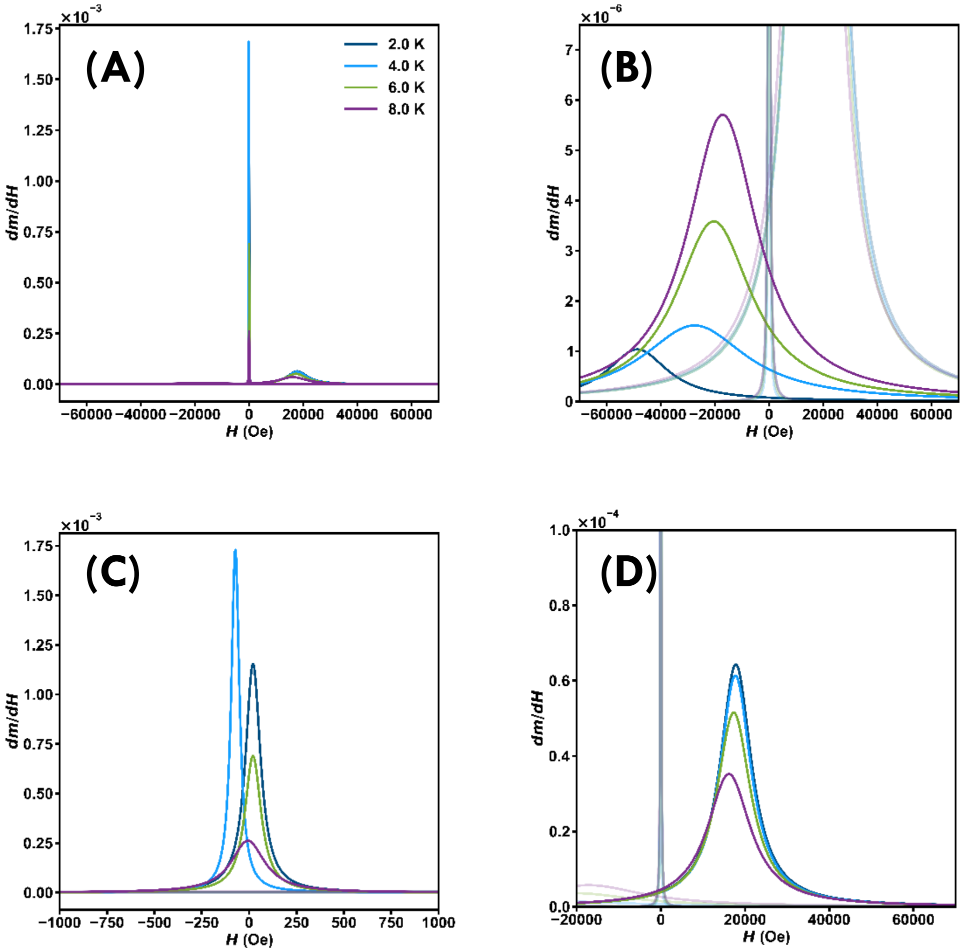

Figure S48. Cauchy fits of isothermal magnetization loops collected at T = 2, 4, 6, and 8 K of **2-Y**. (a) Fits of all three processes. (b) Zoom of process 1. (c) Zoom of process 2. (d) Zoom of process 3.

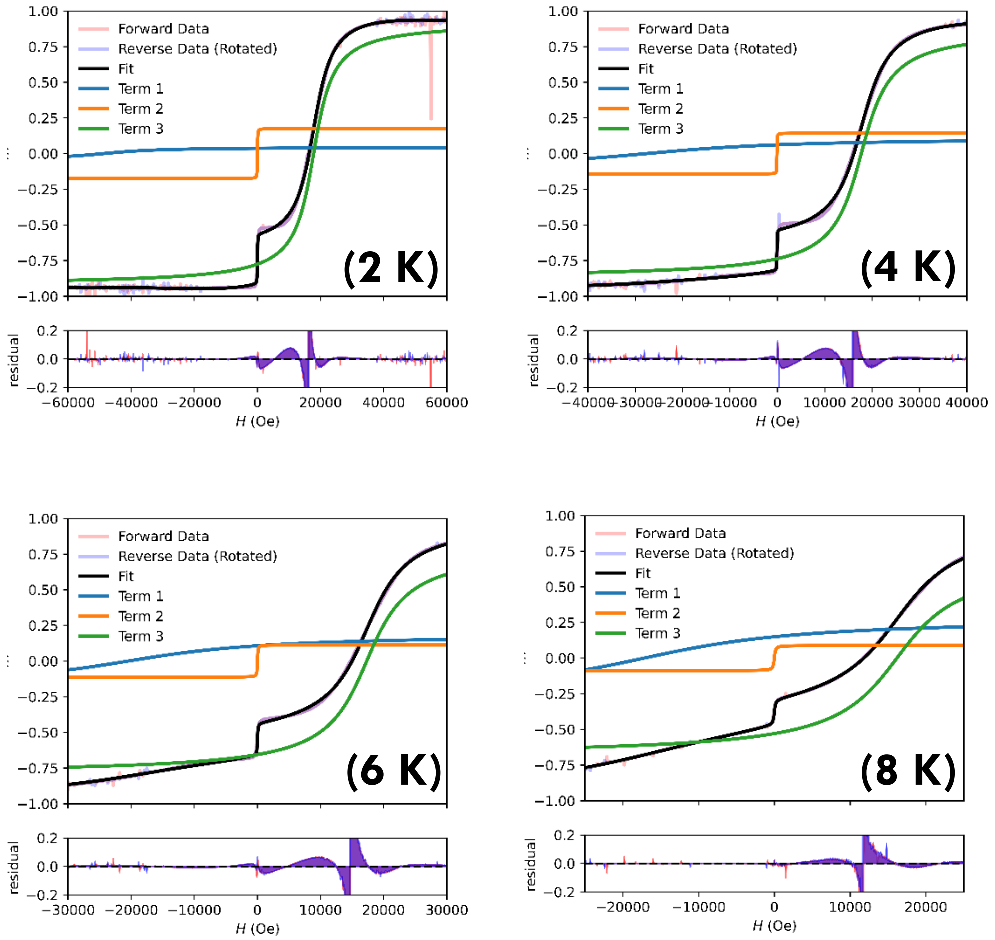

Figure S49. Cauchy fits of isothermal magnetization loops collected at T = 2, 4, 6, and 8 K of **2-Y,** showing contributions of each term (process). Residuals are demonstrated below each graph.

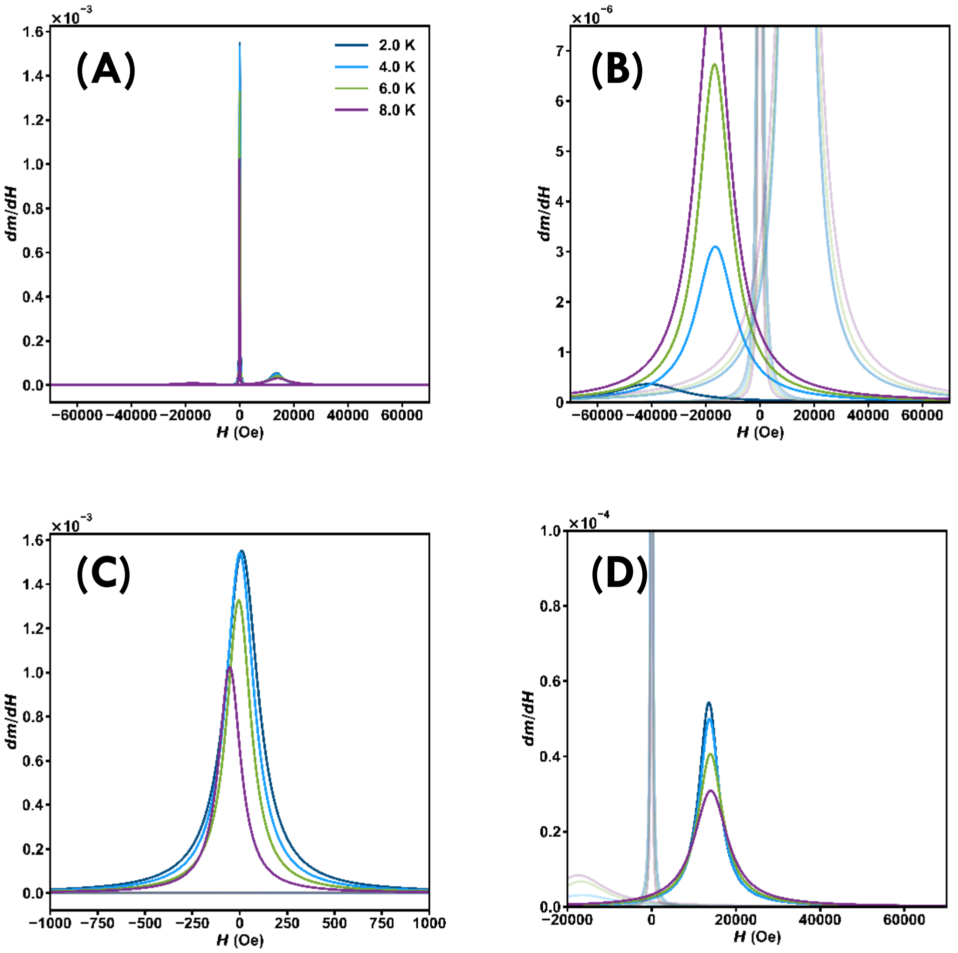

Figure S50. Cauchy fits of isothermal magnetization loops collected at T = 2, 4, 6, and 8 K of **3**. (a) Fits of all three processes. (b) Zoom of process 1. (c) Zoom of process 2. (d) Zoom of process 3.

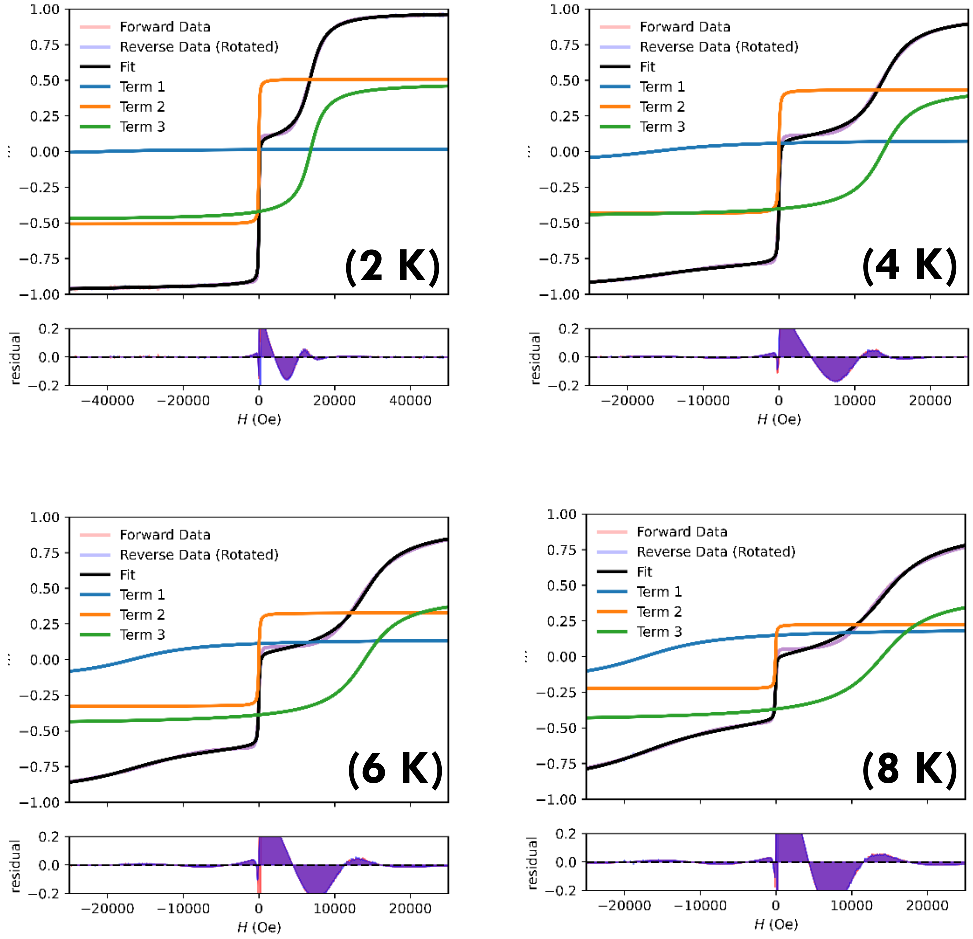

Figure S51. Cauchy fits of isothermal magnetization loops collected at T = 2, 4, 6, and 8 K of **3**, showing contributions of each term (process). Residuals are demonstrated below each graph.

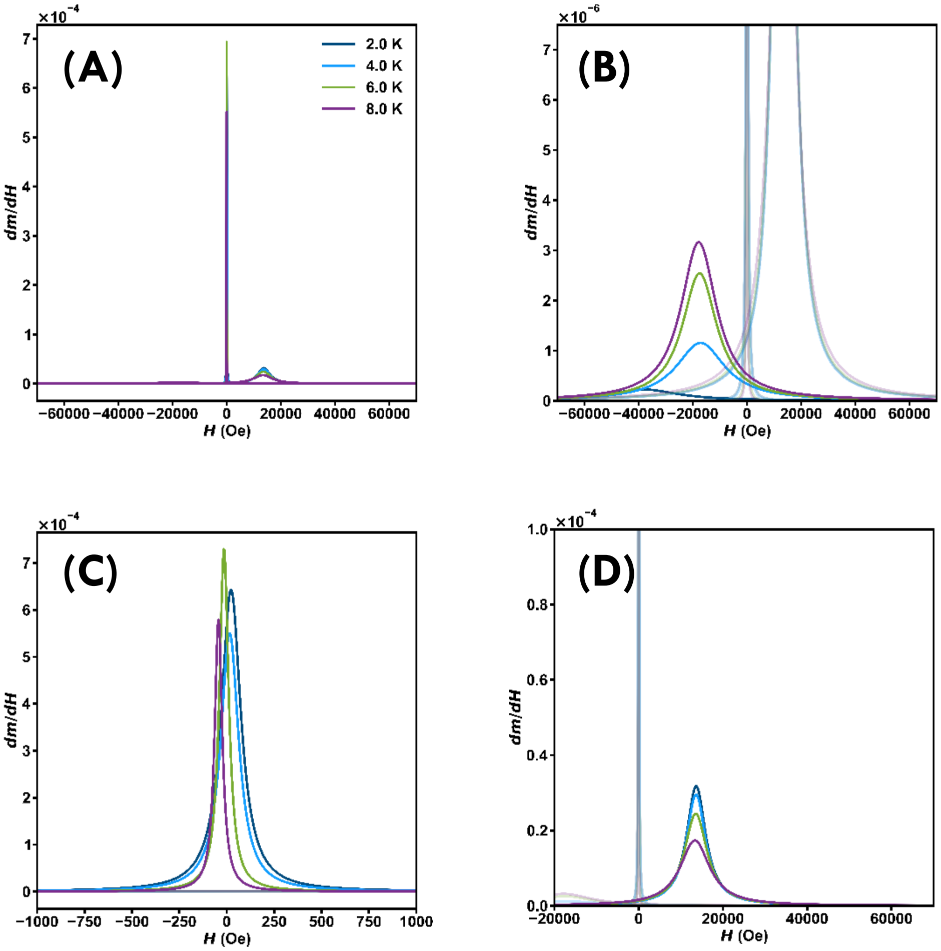

Figure S52. Cauchy fits of isothermal magnetization loops collected at T = 2, 4, 6, and 8 K of **3-Y**. (a) Fits of all three processes. (b) Zoom of process 1. (c) Zoom of process 2. (d) Zoom of process 3.

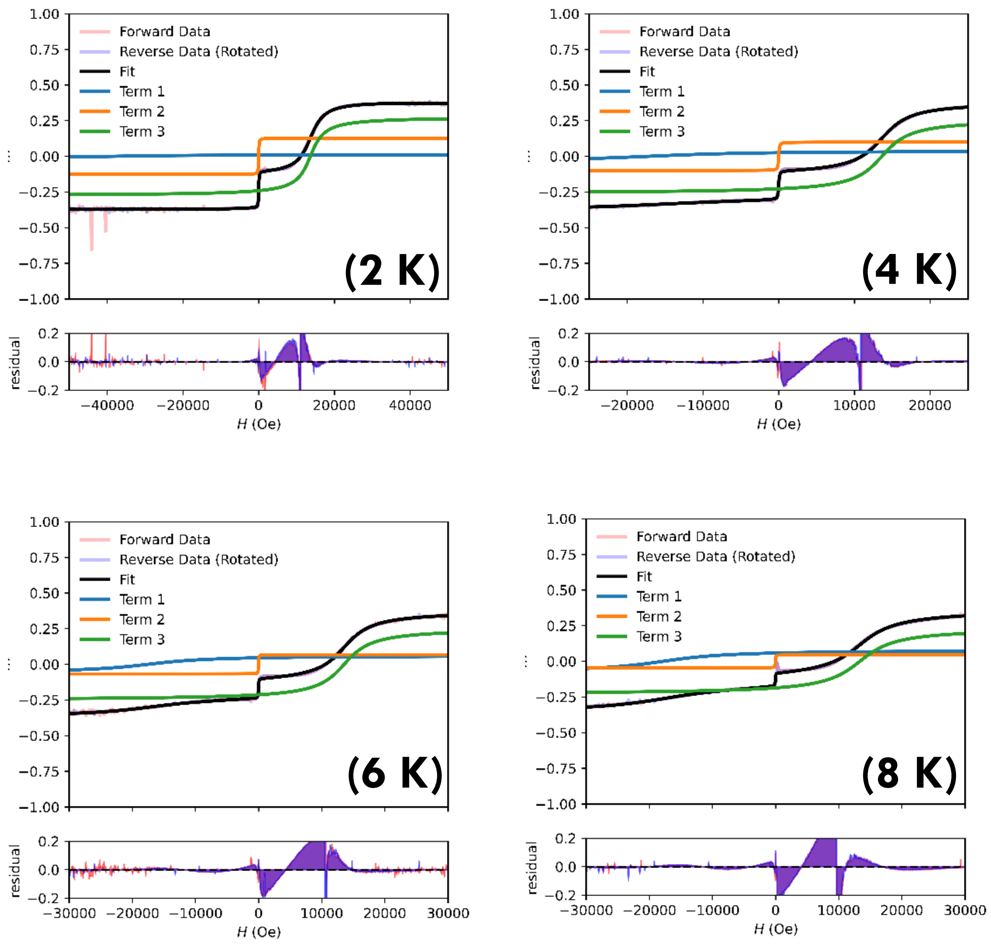

Figure S53. Cauchy fits of isothermal magnetization loops collected at T = 2, 4, 6, and 8 K of **3-Y**, showing contributions of each term (process). Residuals are demonstrated below each graph.

Table S5. Fit parameters arising from Cauchy fitments completed on **1**.

Temp. (K)	M_S Peak 1	M_S error Peak 1	M_S Peak 2	M_S error Peak 2	M_S Peak 3	M_S error Peak 3	H_P Peak 1	H_P error Peak 1	H_P Peak 2	H_P error Peak 2	H_P Peak 3	H_P error Peak 3
2	0.065	0.001	0.096	0.000	0.943	0.001	-47841	550	62	11	24004	7
4	0.096	0.002	0.086	0.001	0.919	0.002	-37779	409	17	11	23923	7
6	0.136	0.002	0.074	0.001	0.862	0.001	-31173	226	-1	13	23694	7
8	0.173	0.001	0.069	0.000	0.775	0.001	-28147	101	-49	9	23075	6
Temp. (K)	γ Peak 1	γ error Peak 1	γ Peak 2	γ error Peak 2	γ Peak 3	γ error Peak 3	Max Height Peak 1	Max Height Peak 2	Max Height Peak 3	Percent Contrib-ution Peak 1	Percent Contrib-ution Peak 2	Percent Contrib-ution Peak 3
2	22697	691	195	12	4592	15	9.17E-07	1.57E-04	6.54E-05	4.3	9.2	86.5
4	24882	612	168	13	4548	16	1.23E-06	1.63E-04	6.43E-05	6.7	8.3	85.0
6	20748	304	120	12	4609	15	2.09E-06	1.95E-04	5.95E-05	10.6	7.4	82.1
8	18001	127	120	10	5018	12	3.05E-06	1.82E-04	4.92E-05	14.9	7.3	77.9

Table S6. Fit parameters arising from Cauchy fitments completed on **1-Y**.

Temp. (K)	M_S Peak 1	M_S error Peak 1	M_S Peak 2	M_S error Peak 2	M_S Peak 3	M_S error Peak 3	H_P Peak 1	H_P error Peak 1	H_P Peak 2	H_P error Peak 2	H_P Peak 3	H_P error Peak 3
2	0.035	0.002	0.019	0.000	0.705	0.002	-52587	1159	-86	12	24849	11
4	0.052	0.001	0.014	0.000	0.695	0.001	-43261	580	-95	4	24770	8
6	0.084	0.002	0.011	0.000	0.661	0.002	-34162	357	-97	9	24502	10
8	0.116	0.001	0.014	0.000	0.600	0.001	-29836	130	-16	13	23796	6
Temp. (K)	γ Peak 1	γ error Peak 1	γ Peak 2	γ error Peak 2	γ Peak 3	γ error Peak 3	Max Height Peak 1	Max Height Peak 2	Max Height Peak 3	Percent Contrib-ution Peak 1	Percent Contrib-ution Peak 2	Percent Contrib-ution Peak 3
2	19119	1272	9	19	4246	23	5.76E-07	5.01E-04	5.29E-05	3.3	2.6	94.1
4	22810	786	5	0	4226	17	7.32E-07	5.52E-04	5.24E-05	5.2	1.8	92.9
6	20212	511	7	18	4279	21	1.32E-06	2.89E-04	4.91E-05	9.2	1.4	89.4
8	18669	171	39	18	4687	13	1.99E-06	1.12E-04	4.07E-05	13.8	2.1	84.2

Table S7. Fit parameters arising from Cauchy fitments completed on **2**.

Temp. (K)	M_S Peak 1	M_S error Peak 1	M_S Peak 2	M_S error Peak 2	M_S Peak 3	M_S error Peak 3	H_P Peak 1	H_P error Peak 1	H_P Peak 2	H_P error Peak 2	H_P Peak 3	H_P error Peak 3
2	0.058	0.002	0.278	0.001	0.726	0.001	-36199	725	81	4	17031	10
4	0.133	0.003	0.239	0.001	0.682	0.002	-19156	435	49	4	17066	10
6	0.185	0.002	0.193	0.001	0.623	0.001	-18204	134	1	4	16846	10
8	0.259	0.001	0.142	0.000	0.558	0.001	-16592	55	-71	3	16006	8
Temp. (K)	γ Peak 1	γ error Peak 1	γ Peak 2	γ error Peak 2	γ Peak 3	γ error Peak 3	Max Height Peak 1	Max Height Peak 2	Max Height Peak 3	Percent Contrib-ution Peak 1	Percent Contrib-ution Peak 2	Percent Contrib-ution Peak 3
2	25118	1047	222	5	4272	19	7.31E-07	3.99E-04	5.41E-05	4.1	27.3	68.5
4	20472	442	185	5	4276	19	2.07E-06	4.11E-04	5.08E-05	10.7	23.9	65.4
6	14031	147	168	5	4716	18	4.21E-06	3.64E-04	4.20E-05	16.9	20.3	62.7
8	13422	62	189	4	6030	15	6.15E-06	2.38E-04	2.94E-05	25.3	15.8	58.9

Table S8. Fit parameters arising from Cauchy fitments completed on **2-Y**.

Temp. (K)	M_S Peak 1	M_S error Peak 1	M_S Peak 2	M_S error Peak 2	M_S Peak 3	M_S error Peak 3	H_P Peak 1	H_P error Peak 1	H_P Peak 2	H_P error Peak 2	H_P Peak 3	H_P error Peak 3
2	0.045	0.002	0.175	0.001	0.923	0.004	-48635	1004	21	7	17808	20
4	0.112	0.003	0.143	0.001	0.878	0.002	-27547	628	-73	10	17707	12
6	0.192	0.002	0.112	0.001	0.796	0.002	-20456	229	20	5	17311	12
8	0.284	0.002	0.088	0.001	0.692	0.002	-17161	135	-6	10	16170	14
Temp. (K)	γ Peak 1	γ error Peak 1	γ Peak 2	γ error Peak 2	γ Peak 3	γ error Peak 3	Max Height Peak 1	Max Height Peak 2	Max Height Peak 3	Percent Contrib-ution Peak 1	Percent Contrib-ution Peak 2	Percent Contrib-ution Peak 3
2	13669	1228	48	10	4568	39	1.04E-06	1.15E-03	6.43E-05	3.2	16.0	80.8
4	23508	781	26	7	4555	24	1.51E-06	1.68E-03	6.13E-05	8.0	13.4	78.6
6	17046	248	52	7	4919	23	3.58E-06	6.89E-04	5.15E-05	15.6	10.9	73.6
8	15832	139	108	11	6257	27	5.71E-06	2.61E-04	3.52E-05	24.6	9.0	66.4

Table S9. Fit parameters arising from Cauchy fitments completed on **3**.

Temp. (K)	M_S Peak 1	M_S error Peak 1	M_S Peak 2	M_S error Peak 2	M_S Peak 3	M_S error Peak 3	H_P Peak 1	H_P error Peak 1	H_P Peak 2	H_P error Peak 2	H_P Peak 3	H_P error Peak 3
2	0.017	0.001	0.508	0.001	0.484	0.001	-41179	996	11	1	13591	10
4	0.084	0.001	0.434	0.001	0.464	0.001	-16506	172	1	1	13742	11
6	0.150	0.001	0.327	0.001	0.463	0.001	-16752	91	-5	2	13959	16
8	0.204	0.002	0.225	0.001	0.466	0.002	-17335	82	-53	4	14043	23

Temp. (K)	γ Peak 1	γ error Peak 1	γ Peak 2	γ error Peak 2	γ Peak 3	γ error Peak 3	Max Height Peak 1	Max Height Peak 2	Max Height Peak 3	Percent Contrib-ution Peak 1	Percent Contrib-ution Peak 2	Percent Contrib-ution Peak 3
2	15273	1404	104	2	2834	18	3.61E-07	1.55E-03	5.43E-05	1.4	51.2	47.4
4	8667	215	90	2	2957	17	3.10E-06	1.53E-03	5.00E-05	8.0	45.0	46.9
6	7111	124	78	3	3626	25	6.73E-06	1.33E-03	4.06E-05	15.3	35.8	48.9
8	7737	114	70	4	4800	39	8.38E-06	1.02E-03	3.09E-05	22.0	26.2	51.8

Table S10. Fit parameters arising from Cauchy fitments completed on **3-Y**.

Temp. (K)	M_S Peak 1	M_S error Peak 1	M_S Peak 2	M_S error Peak 2	M_S Peak 3	M_S error Peak 3	H_P Peak 1	H_P error Peak 1	H_P Peak 2	H_P error Peak 2	H_P Peak 3	H_P error Peak 3
2	0.011	0.001	0.127	0.000	0.276	0.001	-38235	1564	23	4	13651	18
4	0.040	0.001	0.099	0.000	0.262	0.001	-17144	310	16	3	13621	12
6	0.062	0.001	0.067	0.001	0.252	0.001	-17505	223	-14	4	13543	25
8	0.079	0.001	0.044	0.000	0.232	0.001	-17815	128	-44	4	13345	25

Temp. (K)	γ Peak 1	γ error Peak 1	γ Peak 2	γ error Peak 2	γ Peak 3	γ error Peak 3	Max Height Peak 1	Max Height Peak 2	Max Height Peak 3	Percent Contrib-ution Peak 1	Percent Contrib-ution Peak 2	Percent Contrib-ution Peak 3
2	15588	2238	63	5	2767	29	2.25E-07	6.42E-04	3.18E-05	2.2	31.3	66.5
4	10943	359	58	4	2830	19	1.15E-06	5.45E-04	2.95E-05	9.1	25.5	65.4
6	7731	297	29	6	3280	41	2.54E-06	6.94E-04	2.44E-05	15.5	18.3	66.2
8	7971	171	24	5	4260	41	3.16E-06	5.52E-04	1.73E-05	21.5	13.0	65.5

8 REFERENCES

(1) Meihaus, K. R.; Long, J. R. "Magnetic Blocking at 10 K and a Dipolar-Mediated Avalanche in Salts of the Bis(eta(8)-cyclooctatetraenide) Complex [Er(COT)(2)](-)" *J Am Chem Soc* **2013**, *135*, 17952.
(2) Sheldrick, G. M. "Structure determination revisited" *Acta Crystallogr A* **2015**, *71*, S9.
(3) Sheldrick, G. M. "SHELXT - Integrated space-group and crystal-structure determination" *Acta Crystallogr A* **2015**, *71*, 3.
(4) Sheldrick, G. M. "Crystal structure refinement with SHELXL" *Acta Crystallogr C* **2015**, *71*, 3.
(5) Dolomanov, O. V.; Bourhis, L. J.; Gildea, R. J.; Howard, J. A. K.; Puschmann, H. "OLEX2: a complete structure solution, refinement and analysis program" *J Appl Crystallogr* **2009**, *42*, 339.
(6) Bain, G. A.; Berry, J. F. "Diamagnetic corrections and Pascal's constants" *J Chem Educ* **2008**, *85*, 532.
(7) Hilgar, J. D.; Butts, A. K.; Rinehart, J. D. "A method for extending AC susceptometry to long-timescale magnetic relaxation" *Phys Chem Chem Phys* **2019**, *21*, 22302.
(8) Orlova, A. P.; Hilgar, J. D.; Bernbeck, M. G.; Gembicky, M.; Rinehart, J. D. "Intuitive Control of Low-Energy Magnetic Excitations via Directed Dipolar Interactions in a Series of Er(III)-Based Complexes" *J Am Chem Soc* **2022**, *144*, 11316.
(9) Hilgar, J. D., Orlova, A.P., Bernbeck, M.G. 2022.
(10) Aquilante, F.; Autschbach, J.; Baiardi, A.; Battaglia, S.; Borin, V. A.; Chibotaru, L. F.; Conti, I.; De Vico, L.; Delcey, M.; Galvan, I. F.; Ferre, N.; Freitag, L.; Garavelli, M.; Gong, X. J.; Knecht, S.; Larsson, E. D.; Lindh, R.; Lundberg, M.; Malmqvist, P. A.; Nenov, A.; Norell, J.; Odelius, M.; Olivucci, M.; Pedersen, T. B.; Pedraza-Gonzalez, L.; Phung, Q. M.; Pierloot, K.; Reiher, M.; Schapiro, I.; Segarra-Marti, J.; Segatta, F.; Seijo, L.; Sen, S.; Sergentu, D. C.; Stein, C. J.; Ungur, L.; Vacher, M.; Valentini, A.; Veryazov, V. "Modern quantum chemistry with [Open]Molcas" *J Chem Phys* **2020**, *152*.
(11) Galvan, I. F.; Vacher, M.; Alavi, A.; Angeli, C.; Aquilante, F.; Autschbach, J.; Bao, J. J.; Bokarev, S. I.; Bogdanov, N. A.; Carlson, R. K.; Chibotaru, L. F.; Creutzberg, J.; Dattani, N.; Delcey, M. G.; Dong, S. J. S.; Dreuw, A.; Freitag, L.; Frutos, L. M.; Gagliardi, L.; Gendron, F.; Giussani, A.; Gonzalez, L.; Grell, G.; Guo, M. Y.; Hoyer, C. E.; Johansson, M.; Keller, S.; Knecht, S.; Kovacevic, G.; Kallman, E.; Li Manni, G.; Lundberg, M.; Ma, Y. J.; Mai, S.; Malhado, J. P.; Malmqvist, P. A.; Marquetand, P.; Mewes, S. A.; Norell, J.; Olivucci, M.; Oppel, M.; Phung, Q. M.; Pierloot, K.; Plasser, F.; Reiher, M.; Sand, A. M.; Schapiro, I.; Sharma, P.; Stein, C. J.; Sorensen, L. K.; Truhlar, D. G.; Ugandi, M.; Ungur, L.; Valentini, A.; Vancoillie, S.; Veryazov, V.; Weser, O.; Wesolowski, T. A.; Widmark, P. O.; Wouters, S.; Zech, A.; Zobel, J. P.; Lindh, R. "OpenMolcas: From Source Code to Insight" *Journal of Chemical Theory and Computation* **2019**, *15*, 5925.
(12) Kirkpatrick, K. M.; Zhou, B. H.; Bunting, P. C.; Rinehart, J. D. "Quantifying superparamagnetic signatures in nanoparticle magnetite: a generalized approach for physically meaningful statistics and synthesis diagnostics" *Chem Sci* **2023**.
(13) Bunting, P. C.; Rinehart, J. D.; v0.2.0 ed. Zenodo, 2023.
